# A clinically relevant sheep model of orthotopic heart transplantation 24 h after donor brainstem death

**DOI:** 10.1186/s40635-021-00425-4

**Published:** 2021-12-24

**Authors:** Louise E. See Hoe, Karin Wildi, Nchafatso G. Obonyo, Nicole Bartnikowski, Charles McDonald, Kei Sato, Silver Heinsar, Sanne Engkilde-Pedersen, Sara Diab, Margaret R. Passmore, Matthew A. Wells, Ai-Ching Boon, Arlanna Esguerra, David G. Platts, Lynnette James, Mahe Bouquet, Kieran Hyslop, Tristan Shuker, Carmen Ainola, Sebastiano M. Colombo, Emily S. Wilson, Jonathan E. Millar, Maximillian V. Malfertheiner, Janice D. Reid, Hollier O’Neill, Samantha Livingstone, Gabriella Abbate, Noriko Sato, Ting He, Viktor von Bahr, Sacha Rozencwajg, Liam Byrne, Leticia P. Pimenta, Lachlan Marshall, Lawrie Nair, John-Paul Tung, Jonathan Chan, Haris Haqqani, Peter Molenaar, Gianluigi Li Bassi, Jacky Y. Suen, David C. McGiffin, John F. Fraser

**Affiliations:** 1 Critical Care Research Group, The Prince Charles Hospital, Brisbane, QLD Australia; 2 Prince Charles Hospital Northside Clinical Unit, Faculty of Medicine, University of Queensland, Brisbane, QLD Australia; 3 Cardiovascular Research Institute Basel, Basel, Switzerland; 4 Wellcome Trust Centre for Global Health Research, Imperial College London, London, UK; 5 Initiative to Develop African Research Leaders (IDeAL), Kilifi, Kenya; 6 School of Mechanical, Medical and Process Engineering, Faculty of Engineering, Queensland University of Technology, Brisbane, QLD Australia; 7 Department of Anaesthesia and Perfusion, The Prince Charles Hospital, Chermside, QLD Australia; 8 Second Department of Intensive Care, North Estonia Medical Centre, Tallinn, Estonia; 9 Research and Development, Australian Red Cross Lifeblood, Brisbane, QLD Australia; 10 School of Pharmacy and Medical Sciences, Griffith University, Southport, QLD Australia; 11 Department of Cardiac Surgery, Princess Alexandra Hospital, Brisbane, QLD Australia; 12 School of Biomedical Sciences, Faculty of Medicine, University of Queensland, Brisbane, QLD Australia; 13 Department of Pathophysiology and Transplantation, Università Degli Studi di Milano, Milan, Italy; 14 Roslin Institute, University of Edinburgh, Edinburgh, UK; 15 Department of Internal Medicine II, Cardiology and Pneumology, University Medical Center Regensburg, Regensburg, Germany; 16 Department of Physiology and Pharmacology, Section for Anesthesiology and Intensive Care Medicine, Karolinska Institutet, Stockholm, Sweden; 17 Pitié-Salpêtrière University Hospital, Paris, France; 18 The Canberra Hospital Intensive Care, Garran, ACT Australia; 19 Australia National University, Canberra, ACT Australia; 20 Prince Charles Hospital, Brisbane, QLD Australia; 21 Faculty of Health, Queensland University of Technology, Brisbane, QLD Australia; 22 School of Medicine, Griffith University, Southport, QLD Australia; 23 Faculty of Health, School of Biomedical Sciences, Queensland University of Technology, Brisbane, QLD Australia; 24 Institut d’Investigacions Biomèdiques August Pi Sunyer (IDIBAPS), Barcelona, Spain; 25 Cardiothoracic Surgery and Transplantation, The Alfred Hospital, Melbourne, VIC Australia; 26 Monash University, Melbourne, VIC Australia

**Keywords:** Heart transplantation, Brainstem death, Systemic inflammation, Cold static storage, Cardiovascular system, Ischemia, Reperfusion

## Abstract

**Background:**

Heart transplantation (HTx) from brainstem dead (BSD) donors is the gold-standard therapy for severe/end-stage cardiac disease, but is limited by a global donor heart shortage. Consequently, innovative solutions to increase donor heart availability and utilisation are rapidly expanding. Clinically relevant preclinical models are essential for evaluating interventions for human translation, yet few exist that accurately mimic all key HTx components, incorporating injuries beginning in the donor, through to the recipient. To enable future assessment of novel perfusion technologies in our research program, we thus aimed to develop a clinically relevant sheep model of HTx following 24 h of donor BSD.

**Methods:**

BSD donors (vs. sham neurological injury, 4/group) were hemodynamically supported and monitored for 24 h, followed by heart preservation with cold static storage. Bicaval orthotopic HTx was performed in matched recipients, who were weaned from cardiopulmonary bypass (CPB), and monitored for 6 h. Donor and recipient blood were assayed for inflammatory and cardiac injury markers, and cardiac function was assessed using echocardiography. Repeated measurements between the two different groups during the study observation period were assessed by mixed ANOVA for repeated measures.

**Results:**

Brainstem death caused an immediate catecholaminergic hemodynamic response (mean arterial pressure, *p* = 0.09), systemic inflammation (IL-6 - *p* = 0.025, IL-8 - *p* = 0.002) and cardiac injury (cardiac troponin I, *p* = 0.048), requiring vasopressor support (vasopressor dependency index, VDI, *p* = 0.023), with normalisation of biomarkers and physiology over 24 h. All hearts were weaned from CPB and monitored for 6 h post-HTx, except one (sham) recipient that died 2 h post-HTx. Hemodynamic (VDI - *p* = 0.592, heart rate - *p* = 0.747) and metabolic (blood lactate, *p* = 0.546) parameters post-HTx were comparable between groups, despite the observed physiological perturbations that occurred during donor BSD. All p values denote interaction among groups and time in the ANOVA for repeated measures.

**Conclusions:**

We have successfully developed an ovine HTx model following 24 h of donor BSD. After 6 h of critical care management post-HTx, there were no differences between groups, despite evident hemodynamic perturbations, systemic inflammation, and cardiac injury observed during donor BSD. This preclinical model provides a platform for critical assessment of injury development pre- and post-HTx, and novel therapeutic evaluation.

**Supplementary Information:**

The online version contains supplementary material available at 10.1186/s40635-021-00425-4.

## Background

Heart transplantation (HTx) is the gold standard treatment for severe or end-stage cardiac disease patients, but is severely limited by the persistent global shortage of donor hearts [1]. Hearts are predominantly procured from donors sustaining a severe neurologic insult culminating in brain stem death (BSD). The donor heart is exposed to cumulative injury throughout the entire process of HTx which is largely unavoidable with current practices, and contributes to primary graft dysfunction (PGD), the largest cause of early death post-HTx [2–4]. Beyond an ischemic time of 4 h, the probability of PGD increases, a phenomenon aggravated by increasing donor age [5]. Donor hearts are exposed to cold ischemia during preservation via cold static storage (CSS), warm ischemia during engraftment, and subsequent reperfusion injury upon removal of the aortic cross clamp. This unavoidable ischemia-reperfusion injury (IRI) during HTx is detrimental to cardiac allograft function and aggravates PGD [4]. Mitigating the detrimental effects of cumulative injury during HTx is imperative to improving recipient survival post-HTx.

The global donor heart shortage has galvanised preclinical research around understanding BSD-mediated pathophysiology and resultant cardiac dysfunction. Clinical experience with donation after circulatory determination of death as an alternative source for donor hearts is expanding [6–8]. Furthermore, the availability of novel avenues to improve donor heart preservation and ameliorate myocardial IRI associated with HTx is increasing. Normothermic and hypothermic ex vivo perfusion systems have shown promising clinical results, expanding use of ‘marginal’ donor hearts with acceptable post-HTx outcomes, as well as safely increasing ischemic times to overcome logistical limitations [7, 9–12]. Preclinical research is vital to these clinical advances, allowing important in vivo physiological and cellular understanding of injury development and effective treatment. Unfortunately, there are few reported animal HTx models that incorporate donor BSD [13]. These models exhibit significant heterogeneity (BSD length, HTx technique, and ischemic times) and variation in reporting of model components, many of which impact post-HTx outcomes [13]. In addition, existing preclinical models of BSD-HTx are very complex, with significant financial and technical challenges.

We previously profiled the physiological and biological impact of BSD on donors over 24 h [14]. To facilitate future research and investigate novel strategies to increase donor heart availability for HTx, we sought to extend this published model and incorporate HTx. Consequently, we aimed to develop a clinically relevant sheep model of HTx, incorporating 24 h donor BSD, heart preservation by CSS, bicaval orthotopic HTx, and 6-h follow-up after successful weaning from CPB.

## Methods

### Experimental design

**Fig. 1:**
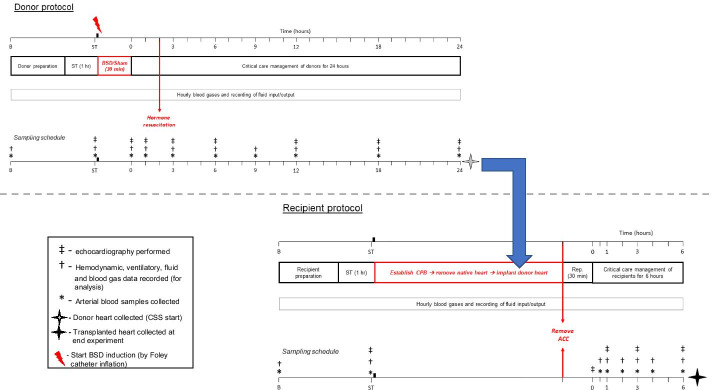
Experimental timeline for donor BSD and subsequent HTx*.* All sheep were approved for use by the facility veterinarian after a comprehensive health check. Prior to allocation, experimental sheep were matched for weight and blood compatibility [17], then paired and randomly assigned to either donor sham (control without neurological injury) or BSD groups (*n* = 4/group). Sheep within each pair were then randomly allocated as an experimental donor or recipient. Following standard instrumentation procedures, donor animals were rested for 1 h prior to induction of BSD/sham (ST). Following confirmation of BSD (T0 in the donor), donors were administered hormone resuscitation 2 h thereafter (T2), and monitored for 24 h in intensive care settings, prior to donor heart procurement and heart preservation by cold static storage (CSS). In donors, at baseline (B), stabilisation (ST), upon confirmation of BSD/sham (T0), and 1, 3, 6, 9, 12, 18 and 24 h thereafter, arterial blood was collected for determination of full blood counts, plasma catecholamines, inflammatory markers and cardiac troponin I. Periodic epicardial echocardiography was performed to measure cardiac function at all aforementioned timepoints in donor (except baseline). The recipient was prepared and underwent instrumentation procedures prior to establishment of CPB. The donor heart was implanted into the recipient, and the cardiac allograft was reperfused (Rep.) for 30 min prior to any attempt to wean from CPB (ACC: aortic cross-clamp). After successful weaning from CPB (T0 in recipient), the recipient was followed up for 6 h and then euthanized. In recipients, arterial blood was collected at baseline, stabilisation, and 0.5, 1, 2, 3, 4 and 6 h after weaning from CPB (T0) for determination of full blood counts, plasma catecholamines, inflammatory markers and cardiac troponin I. In recipient animals, echocardiography was performed at stabilisation, T0 and 1, 3 and 6 h thereafter

Donor sheep were either exposed to 24 h brain death (BSD; *n* = 4), using previously published methods [14] or not exposed to BSD (sham; *n* = 4, not exposed to neurological injury). Following confirmation of donor BSD (or sham), critical care management was provided for 24 h. The donor heart was then procured and preserved by CSS. Healthy recipient was then prepared, cardiopulmonary bypass (CPB) established, and the native heart removed. The preserved donor heart was implanted using the bicaval orthotopic HTx technique [15]. The recipient was then weaned from CPB and monitored for up to 6 h (Fig. 1).

### Animals and experimental group selection

Sixteen female merino crossbred sheep (Ovis aries, first cross ewes, 1–3 years in age), body weight 46.9 ± 5.8 kg, were approved for use by the facility veterinarian after a comprehensive health check. Animals were housed in flocks on large institutional paddocks with grass, and free access to shade and ad libitum water. One week prior to experimentation, sheep were transferred to the animal facility to acclimate, with ad libitum food and water. Animal studies were undertaken at the Queensland University of Technology (QUT) Medical Engineering Research Facility in Brisbane (March 2017–December 2018). Animal ethics was approved by the QUT Animal Ethics Committee (AEC) (16000001109) and ratified by the University of Queensland AEC (QUT/393/17/QUT). All experiments were performed in accordance with the National Health and Medical Research Council (NHMRC) *Australian Code of Practice for the Care and Use of Animals for Scientific Purposes 8th Edition 2013* and the *Animal Care and Protection Act 2001 (QLD),* and complied with the ARRIVE Guidelines [16]*.* Prior to group allocation, experimental sheep were matched for weight and blood compatibility [17], then paired and randomly assigned to either donor sham or BSD groups (*n* = 4/group). Sheep within each pair were then randomly allocated as an experimental donor or recipient.

### Animal preparation (both donors and recipients)

All sheep were fasted overnight, and in the morning brought into the surgical theatre. Five mL of lidocaine 2% was administered into the neck skin, the external jugular veins were cannulated, and a baseline venous blood sample collected. The animal was then induced with midazolam (0.5 mg/kg) and propofol (2.5–3 mg/kg), and antibiotics were administered (gentamycin 80 mg and cefazolin 2 g). The animal was then intubated, and placed on the operating table in right lateral (donors) or supine position (recipients). Electrocardiography (ECG), oxygen saturation (SpO_2_) and end-tidal carbon dioxide (ETCO_2_) were continuously monitored. The animal was connected to a mechanical ventilator (Hamilton-G5, Hamilton Medical, Switzerland) set as follows: tidal volume 8 ml/Kg, positive end expiratory pressure (PEEP) 5 cmH_2_O, respiratory rate (RR) adjusted to maintain arterial partial pressure of carbon dioxide (PaCO_2_) at 40 ± 4 mmHg, and inspiratory fraction of oxygen (FiO_2_) adjusted to maintain arterial partial pressure of oxygen (PaO_2_) > 60 mmHg. Anaesthesia and analgesia were maintained with continuous infusions of fentanyl (10–30 μg/kg/h), midazolam (1–3 mg/kg/h) and ketamine (4–12.5 mg/kg/h). All animals received an initial intravenous (i.v.) bolus of 0.25–1 L Hartmann’s solution (guided by static parameters of fluid responsiveness, echocardiography and lactate levels), followed by a continuous infusion (1 mL/kg/h). Gastric and oropharyngeal secretions were continuously suctioned. Potassium chloride (10–30 mmol/L per h) was continuously infused to maintain serum potassium at 4.0–4.5 mmol/L. The femoral artery was cannulated for continuous arterial blood pressure monitoring and blood sampling. A 7.5 Fr pulmonary artery catheter was inserted for continuous cardiac output monitoring. A urinary catheter was then inserted for urine output monitoring and sampling. Throughout the course of the experiment (both donors and recipients), intravascular crystalloid and vasoactive support were provided to maintain hemodynamic stability, aiming for a mean arterial pressure (MAP) of ≥ 65 mmHg, heart rate (HR) 60–110 bpm, and urinary output of ≥ 0.5 mL/kg/h. Specific additional consumable details for the methodology presented are provided in Additional file 1: Table S1.

### Donor-specific preparation and critical care management

Following procedures outlined above, donors were then placed in right lateral position, vecuronium (0.2 mg/kg i.v.) was administered, and a mini left thoracotomy was performed through the upper border of the 5th rib (Additional file 1: Fig S1) to cannulate the azygos vein for coronary sinus blood sampling.

The skull was then prepared as previously described [14]. Briefly, a midline incision on the skull exposed the sagittal and lambdoid sutures (Additional file 1: Fig S2). A 4.5-mm left burr was drilled 1.5 cm from and parallel to the sagittal and lambdoid sutures, and a 20-G intravenous cannula inserted and secured to monitor intracranial pressure (ICP). A second burr hole (6 mm) was created in the same position, but on the contralateral side of the first burr hole (right), and a 16 Fr Foley catheter (30 mL balloon) was placed in the burr hole. Following completion of aforementioned procedures, donor animals were rested for 1 h (end of 1 h rest = stabilisation time point) to achieve vital signs stability, as corroborated by MAP: 65–100 mmHg, HR: 60–110 bpm, central body temperature: 37.0–38.5 °C and normalisation of blood gas parameters (PaCO_2_: 40 ± 4 mmHg, PaO_2_: 80–100 mmHg, potassium: 4.0–4.5 mmol/L, and calcium > 1.1 mmol/L).

Then, in BSD donors, brain injury was developed by intra-cerebral Foley catheter inflation with 10 mL sterile water every 5 min (to a maximum of 60 mL) over 30 min [14]. In case of ST-segment elevation due to catecholaminergic response, 5 mg of i.v. metoprolol was administered every 2–3 min until ST-segment normalization. BSD was confirmed by high ICP and continuous negative cerebral perfusion pressure, loss of hemodynamic response to ongoing Foley catheter balloon inflation, loss of pupillary and corneal reflexes, and an absence of coughing reflex [14]. For sham donors, the Foley catheter was inserted, but not inflated, and animals rested for 30 min thereafter. Time 0 in the donor was designated at confirmation of BSD (using parameters listed above), or end of 30-min additional monitoring for sham donors. All donors were monitored for 24 h from Time 0.

Donor critical care management: Noradrenaline (0.01–0.4 µg/kg/min), dopamine (1–20 µg/kg/min) and/or crystalloid challenges were administered to maintain MAP ≥ 65 mmHg; arterial blood gases and electrolytes were measured hourly (Radiometer ABL-825 analyser, Copenhagen, Denmark) and potassium and calcium supplemented accordingly to maintain potassium 4.0–4.5 mmol/L and calcium > 1.1 mmol/L; ventilatory settings were adjusted to maintain PaCO_2_ at 40 ± 4 mmHg and PaO_2_ > 60 mmHg (as described in ‘Animal preparation’ above); desmopressin (i.v. 4 μg bolus) was administered if urine output consistently exceeded 200 mL in the two proceeding hours. Two hours after BSD confirmation, donors received i.v. methylprednisolone 15 mg/kg, triiodothyronine 4 μg bolus and 3 μg/h thereafter, and vasopressin 1.2 IU/h. The Foley catheter balloon was additionally inflated (10 mL of saline) after methylprednisolone administration to ensure absence of hemodynamic response and residual neurological function (BSD donors only). Sham donors received the same doses of methylprednisolone and triiodothyronine, without vasopressin. Anaesthesia and analgesia were maintained with continuous infusions of fentanyl (10–30 μg/kg/h), midazolam (1–3 mg/kg/h) and ketamine (4–12.5 mg/kg/h). However, in BSD donors, those infusion rates were reduced to trivial levels following BSD confirmation. Donors received continuous infusions of Hartmann’s (1 mL/kg/h) throughout the experiment and fluid boluses as necessary, guided by static parameters of fluid responsiveness, echocardiography and lactate levels.

Following 24 h monitoring, donors were placed supine and a median sternotomy was performed. A purse-string suture was placed in the ascending aorta for the cardioplegic needle (Additional file 1: Fig S3). After full heparinisation, the superior vena cava was clamped, and the inferior vena cava was divided extra-pericardially to exsanguinate the donor. The ascending aorta was clamped and ice-cold St Thomas’s cardioplegia was infused into the aortic root (20 mL/kg). The donor heart was then explanted by dividing the superior vena cava, pulmonary veins, ascending aorta and main pulmonary artery. Following explantation, the donor heart was preserved in an organ bag containing 1-L ice-cold St Thomas’s cardioplegia, and placed in an additional organ bag containing ice slush (0.9% Saline). The organ bag was placed in an ice cooler containing ice slush for approximately 100 min (times outlined below).

### Recipient-specific preparation, orthotopic heart transplantation, and critical care management

All recipients were prepared and instrumented as stated above (‘Animal preparation’). Recipients were then rested for 1 h (end of 1 h rest = stabilisation time point) to achieve vital signs stability (as outlined above for donors). Standard experimental settings for monitoring both donor and recipient (Additional file 1: Fig S4), standard surgical settings (Additional file 1: Fig S5) and additional surgical details (‘Orthotopic HTx procedures’) are provided in the online supplement. The recipient was placed in supine position, paralysed with vecuronium (0.2 mg/kg i.v.), and the chest opened via median sternotomy. Purse-string sutures were placed in the ascending aorta, and caval tapes were placed around the superior and inferior vena cava. The cardiopulmonary bypass (CPB) circuit was primed with 2–3 units of cross-matched ovine packed red blood cells (prepared as previously described [17]). After full heparinisation (100–300 U/kg, to achieve activating clotting time > 400 s), CPB was established with a single aortic cannula and bicaval cannulation, and perfusate temperature reduced to a core temperature of 32 °C. Fluid infusions were stopped upon CPB commencement.

Once CPB was established and the recipient heart removed, standard orthotopic HTx was performed. Anastomoses were performed in the following order: left atrium, inferior vena cava, superior vena cava, pulmonary artery, ascending aorta. After completion of the pulmonary artery anastomosis, amiodarone (150 mg), lignocaine (50 mg), MgSO_4_ (10 mmoL) and methylprednisolone (250 mg) were administered intravenously, and a 7.5 Fr pulmonary artery catheter was inserted for continuous cardiac output monitoring (prior to completion of aortic anastomoses). Fluid infusions recommenced once the animal was rewarmed to 37.0 °C. Following completion of all anastomoses, de-airing was performed with a needle vent in the ascending aorta and the aortic cross-clamp removed. If ventricular fibrillation occurred, internal defibrillation was performed (10–20 Joules), and additional amiodarone (150–300 mg) and lignocaine (50–100 mg) were administered as needed. Cold ischemic time was defined as the time from donor heart placement into an ice cooler until it was removed (Sham: 97.3 ± 8.8 min, BSD: 101.8 ± 12.9 min, *p* = 0.63); total ischemic time was the total time between aortic cross-clamping in the donor, up to removal of the aortic cross-clamp in the recipient (Sham: 158.8 ± 7.4 min, BSD: 171.5 ± 15.5 min, *p* = 0.49). The cardiac allograft was then perfused for 30 min, before attempting to wean from CPB.

Venous return to the pump was ceased when the following criteria were achieved: spontaneous or pacing HR of 80 to 90 beats/minute; MAP ≥ 60 mmHg without substantial vasoactive support (dopamine < 5 μg/kg/min, adrenaline/noradrenaline < 0.1 μg/kg/min, or vasopressin at any dose); adequate ventricle filling as observed using epicardial echocardiography. Total bypass time was defined as time that the recipient was placed on bypass to successful weaning from bypass (Sham: 164.0 ± 32.3 min, BSD: 129.25 ± 10.6 min, *p* = 1.00). Successful separation from CPB was defined as Time 0 in the recipient. Following CPB separation, the CPB cannulae were removed and cannulation sites closed. Residual blood in the extracorporeal circuit reservoir and venous tubing was prepared for transfusion back to the recipient through the central line as required. Anticoagulation was reversed with 25 mg of protamine. Recipient animals were followed up for 6 additional hours following successful separation from CPB. Investigators applied critical care management principles reported in earlier paragraphs (see “Donor-specific preparation and critical care management”) to achieve clinical stability, with additional adrenaline (0.01–1.5 µg/kg/min) or vasopressin (0.5–4 IU/h) as required.

### Data monitoring and acquisition

Hemodynamic parameters were captured at 1 kHz on a 16-channel PowerLab data acquisition system (model ML880) recorded with Labchart 7 (AD Instruments, Bella Vista, Australia). Central venous pressure, MAP, pulmonary artery pressure, ICP, ECG, HR and SpO_2_ and ETCO_2_ were continuously monitored (Marquette Solar 8000, GE Healthcare, Chicago, ILL, USA). Continuous cardiac output, mixed venous oxygen saturation (SvO2), stroke volume, and systemic vascular resistance index were monitored using Vigilance II Monitor (Edward Lifesciences, Irvine, CA, USA). The Hamilton-G5 ventilator was used to record ventilatory parameters at 100 Hz using custom software. Body surface area (BSA) was calculated using the following equation, BSA = 0.094 × (body weight in kg)^0.67^ [18]. As a measure of hemodynamic performance, vasopressor dependency index (VDI) was computed as previously described (VDI = (dopamine + dobutamine + noradrenaline × 100 + adrenaline × 100)/MAP, all doses in μg/kg/min) [19], to determine the amount of vasoactive support that was required to maintain adequate MAP. Animals (both donors and recipients) were vigilantly and continuously monitored by trained clinical staff throughout the course of the experiment. Blood gases and fluid balance were checked and recorded hourly (or more frequently as required), and results used to manage animals accordingly (as outlined above). In donors, hemodynamics, ventilation, blood gases, and fluids were recorded at baseline, stabilisation, upon confirmation of BSD/sham (T0), and 1, 3, 6, 9, 12, 18 and 24 h thereafter. In recipients, these parameters were recorded at baseline, stabilisation, and 0.5, 1, 2, 3, 4 and 6 h after weaning from CPB (T0). Blood gases and fluids were recorded using a tablet (iPad, Apple, Cupertino) connected to an online database with automatic recording.

### Two-dimensional echocardiography and analysis

Epicardial echocardiography was performed through an X5-1 transducer with a spacer connected to an IE-33 ultrasound scanner (Philips, Bothell, WA, USA). In donors, echocardiography was performed at stabilisation, T0 (upon BSD/sham corroboration), 1, 3, 6, 12, 18 and 24 h thereafter. In recipients, echocardiography was performed at baseline, T0 (successful weaning from CPB), and 1, 3 and 6 h thereafter. Three-beat ECG-gated loops in the conventional parasternal short axis (PSAX) view were obtained. Conventional apical views could not be obtained, due to anatomic constraints in accessing the apex related to the short thoracotomy or sternotomy. All images were transferred to a separate workstation and analysed offline by an experienced echocardiographer using TomTec-Arena (TomTec imaging Systems GMBH, Unterschleim, Germany). Automated Functional Imaging (AFI) was applied to appropriate echo-loops in the PSAX view. Speckle tracking was visually assessed for tracking accuracy and the end-diastolic timing marker manually adjusted if required. Data collected were end-diastolic area (EDA, cm^2^), end-systolic area (ESA, cm^2^), fractional area change (FAC, %), and global radial strain (GRS, %). FAC was defined as the EDA minus the ESA divided by the EDA. GRS was defined as the strain derived from the whole thickness of the myocardium in the radial direction.

### Blood sample collection, processing, and analysis (full blood counts, biochemistry and catecholamines)

In donors, at baseline, stabilisation, upon confirmation of BSD/sham (T0), and 1, 3, 6, 9, 12, 18 and 24 h thereafter, arterial blood was collected. In recipients, arterial blood was collected at baseline, stabilisation, and 0.5, 1, 2, 3, 4 and 6 h after weaning from CPB (T0). Blood samples were centrifuged twice (3000×*g*, 15 min, 4 °C) to obtain platelet poor plasma, and stored at − 80 °C. Whole blood (EDTA) and plasma samples were externally assessed by a veterinary diagnostics laboratory (IDEXX Laboratories, Brisbane, Australia) to determine full blood counts, biochemistry and plasma free catecholamines (metanephrine and normetanephrine). Stored plasma samples were assessed in-house to determine levels of inflammatory cytokines and cardiac injury markers (details below).

Independent external assessment of hematological profiles were analysed on a Sysmex XT2000i-V hematology analyser, and blood biochemistry was determined using a Beckman Coulter AU680 ISE chemistry analyser as per standard manufacturers protocols [20, 21]. Plasma free metanephrine and normetanephrine were measured by an automated online Solid Phase Extraction (SPE) coupled to a UPLC chromatographic system [22–24]. Plasma samples were diluted 1:1 with deuterated internal standards in a zinc sulfate/acetonitrile solution in 2 mL 96-well plates. After centrifugation, an aliquot of sample was injected into the online SPE equipped UPLC–MS/MS system. The purified extracts were eluted from the Mass Track Oasis WCX OSM cartridges onto the ACQUITY UPLC BEH Amide column, utilising a gradient of 100 mM ammonium formate, pH 3.0 and acetonitrile. Quantification was achieved by monitoring two transitions for each analyte on a Waters Xevo TQD mass spectrometer (Waters Corporation, MA, USA). The assay time between injections was 4.5 min. The analytical range of the assay was up to 100,000 pmol/L. The limit of quantitation (LOQ) with a CV of 20% was 20 pmol/L for all analytes. The inter-run imprecision across 3 levels for the analytes were all < 8%.

### Inflammatory and cardiac injury markers

The concentrations of EDTA plasma interleukin (IL)-6, IL-8, IL-10, and IL-1β were quantified using in-house developed ELISAs according to previously published methods [25]. Tumour necrosis factor alpha (TNFα; Cat# EBTNF, Invitrogen), big endothelin-1 (BET-1; Cat# BI-20082H, United Bioresearch) and high sensitivity cardiac troponin I (cTnI; Cat# CTNI-9-HSP, Life Diagnostics) levels were determined in plasma using commercial kits as per manufacturer’s instructions [14, 26]. Accuracy of all ELISA assays were confirmed using quality control samples, with inter-and intra-plate variability < 10%.

### Statistical analysis

Data in figures and tables is represented as mean ± standard error of the mean (SEM). Repeated measurements between the two different groups were assessed by ANOVA for repeated measures and reported as *F* values according to the sphericity of the data. Top *F* and *p* values on each graph represent effect of time, and bottom *F* and *p* values represent differences between BSD vs. sham over time (interaction). Continuous variables were compared with Mann–Whitney *U* test, and categorical variables using Pearson Chi-square test. All hypothesis testing was two-tailed and *p*-value of less than 0.05 was considered statistically significant. All statistical analyses were performed using SPSS 27 for Windows (SPSS Inc, Chicago, Illinois, US).

## Results

### Baseline animal characteristics

No significant differences were observed between donors and recipients in age, weight, body surface area (BSA) or body temperature (Table 1).

**Table 1:** Baseline animal characteristics for donors and recipients

	Donors		Recipients	
	SHAM (*n* = 4)	BSD (*n* = 4)	SHAM (*n* = 4)	BSD (*n* = 4)
Age	1–3 years			
Weight (kg)	45.8 (3.5)	47.4 (9.3)	48.0 (6.7)	47.0 (5.0)
BSA (m^2^)	1.2 (0.1)	1.23 (0.2)	1.3 (0.1)	1.2 (0.1)
Temperature (°C)	37.7 (0.7)	37.8 (0.9)	37.5 (0.5)	37.2 (0.8)

All data are expressed as mean (standard deviation). *BSA* body surface area

### BSD-mediated hemodynamic and inflammatory responses

**Fig. 2:**
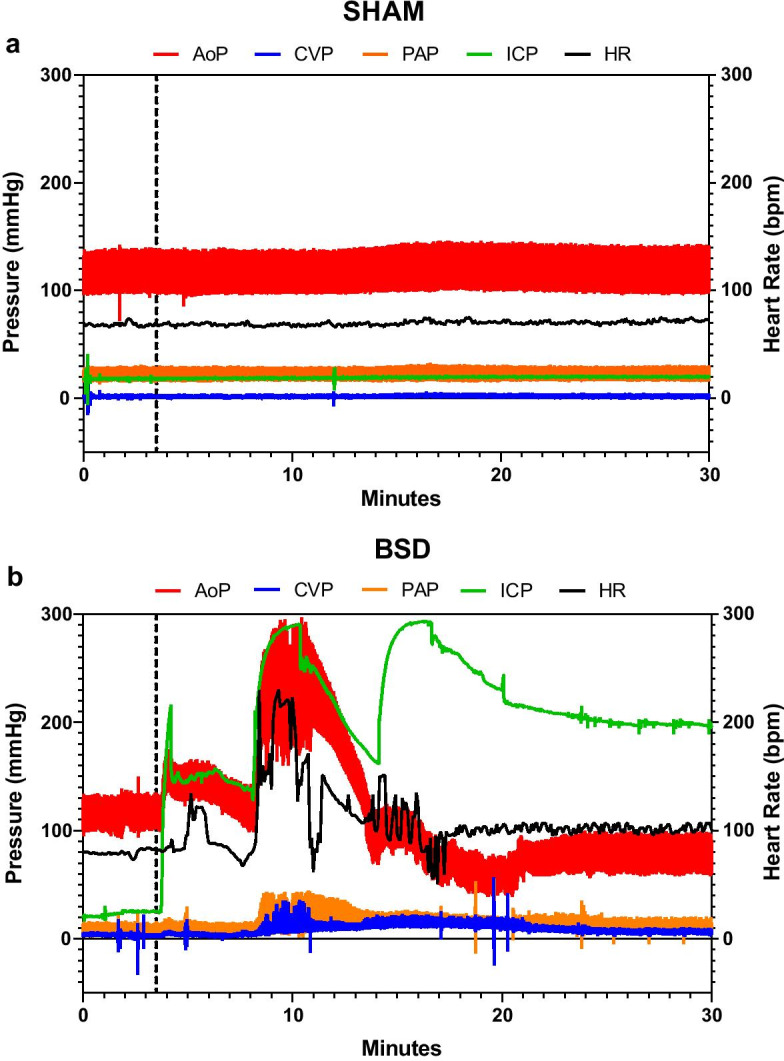
Representative hemodynamic changes during BSD induction (before confirmation of BSD). Changes in aortic pressure (AoP), central venous pressure (CVP), pulmonary artery pressure (PAP), intracranial pressure (ICP) and heart rate (HR) in sham (**a**) and BSD donors (**b**) during BSD/sham induction. Dotted line in **a** represents beginning time-matched rest for sham donors, and commencement of Foley catheter inflation in **b** for BSD donors

**Fig. 3:**
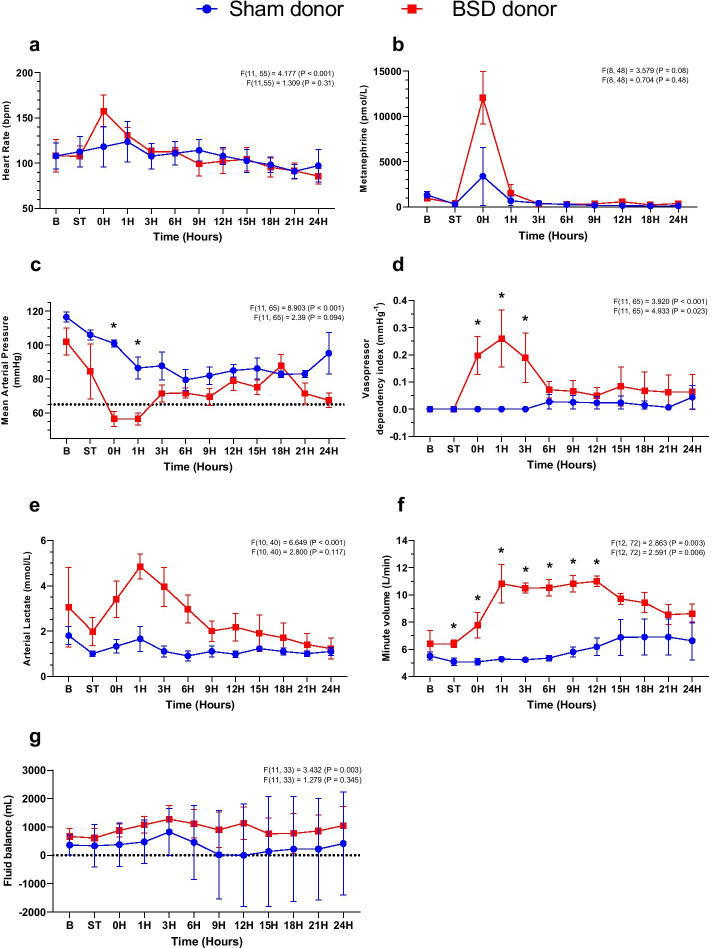
Donor hemodynamics following confirmation of BSD*.* Changes in **a** heart rate (bpm); **b** plasma metanephrine (pmol/L); **c** mean arterial pressure (mmHg); **d** vasopressor dependency index (mmHg^−1^); **e** arterial lactate (mmol/L); **f** minute volume (L/min); and **g** fluid balance (mL) following confirmation of donor BSD (or sham). Data are mean ± SEM, *n* = 4/group. Top *F* and *p* values on each graph represent effect of time, and bottom *F* and *p* values represent differences between BSD vs. sham over time. *B* Baseline, *ST* stabilisation 1 h following completion of instrumentation procedures in donor, 0H—confirmation of BSD

During BSD development, ICP rose significantly in comparison to sham (Sham: 36.3 ± 1.4; BSD: 280.3 ± 19.4 mmHg, Additional file 1: Table S2), coupled with an immediate catecholaminergic response demonstrated by significant elevations in HR and aortic pressure (Fig. 2b). At the time of BSD confirmation (T0), HR (Fig. 3a) and plasma metanephrine (Fig. 3b; Sham: 3379.3 ± 2777.3; BSD: 12,045 ± 2514.3 pmol/L) significantly increased. BSD significantly decreased MAP (Fig. 3c), requiring management with vasopressors (Fig. 3d). Arterial lactate was significantly increased and sustained following BSD development (Fig. 3e; Sham: 1.7 ± 0.5; BSD: 4.9 ± 0.5 mmol/L). Post-BSD, minute volume doubled (Fig. 3f; Sham: 5.3 ± 0.1; BSD: 10.8 ± 1.2 L/min), and there was a tendency towards positive fluid balance. Conversely, in sham animals, the mean fluid balance decreased to zero after 12 h, but slightly increased thereafter (Fig. 3g).

**Fig. 4:**
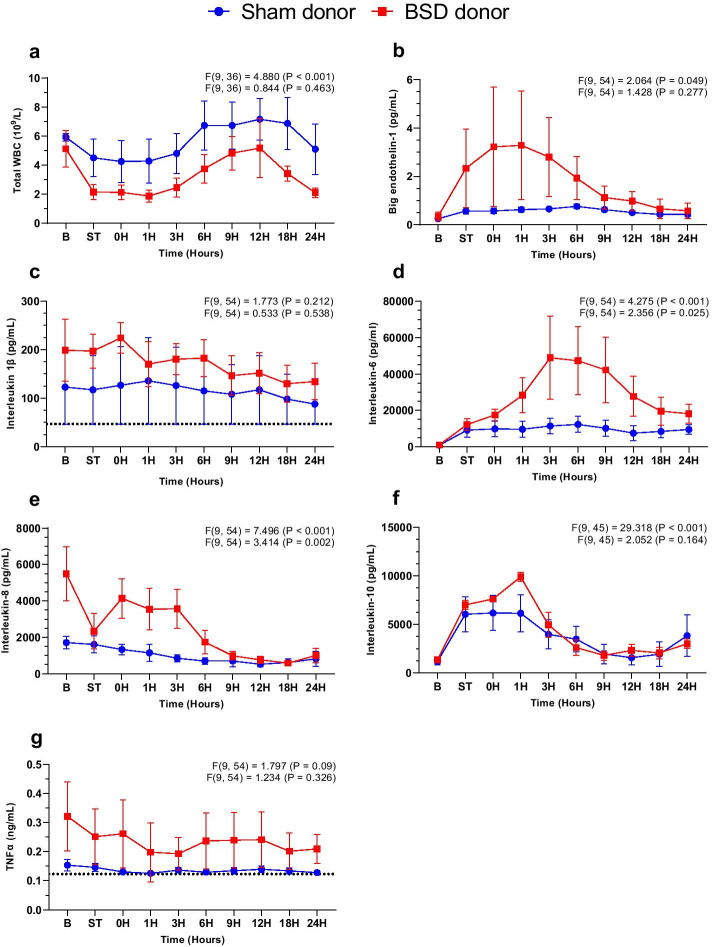
Systemic inflammation in donor animals. Changes in plasma a) total white blood cells (WBC, 10^9^/L); b) big endothelin-1 (BET-1, pg/mL); c) interleukin 1β (pg/mL); **d** interleukin-6 (pg/mL); **e** interleukin-8 (pg/mL); **f** interleukin-10 (pg/mL); and **g** tumour necrosis factor alpha (TNFα, ng/mL) following confirmation of donor BSD (or sham). Data are mean ± SEM, *n* = 4/group. Top *F* and *p* values on each graph represent effect of time, and bottom *F* and p values represent differences between BSD vs. sham over time. *B* Baseline, *ST* stabilisation 1 h following completion of instrumentation procedures in donor, 0H—confirmation of BSD. Dotted lines on **c** and **g** represent the lowest detectable limit of the respective cytokine ELISA

Twenty-four hours after BSD onset, there were no statistically significant differences between sham and BSD donors in the aforementioned parameters, except for MAP that remained lower for BSD donors. However, VDI became similar between groups. Arterial inflammatory markers BET-1 (Fig. 4b), IL-6 (Fig. 4d), and IL-8 (Fig. 4e) increased significantly post-BSD, which settled and was comparable to sham after 24 h. No changes between sham and BSD were observed for total white blood cell counts, IL-1β, IL-10, and TNFα (Fig. 4), despite fluctuation over time. Hemodynamic and ventilatory parameters, blood results and vasoactive use during the course of the study at baseline, T0, T12 and T24 in donor animals are available in Additional file 1: Table S2.

### Heart preservation and transplantation

There were no significant differences in cold ischemic, total ischemic or CPB times between groups (outlined above in “Methods”). Following CSS and orthotopic HTx, all hearts were successfully weaned from CPB, and seven out of eight recipients survived to 6 h post-HTx (one animal receiving a sham donor heart ended 2 h post-HTx). A trend toward higher applied defibrillation energy in sham animals was found to restart the heart following reperfusion (Table 2).

**Table 2:** Transplant outcomes for Sham vs. BSD groups

	SHAM (*n* = 4)	BSD (*n* = 4)	*p* value
Applied defibrillation energy (Joules)	397.5 ± 199.67 J	43.33 ± 19.05 J	0.057
# Successfully weaned from CPB	4	4	
Survival to 6 h post-HTx	3/4	4/4	

Data are means ± SEM, *n* = 4/group

**Fig. 5:**
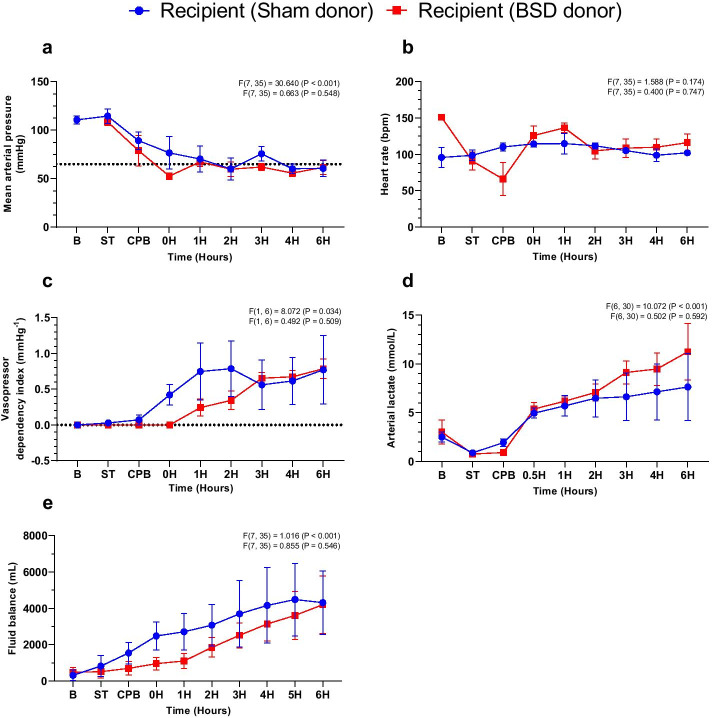
Recipient hemodynamics following CSS and HTx. Changes in **a** mean arterial pressure (mmHg); **b** heart rate (bpm); **c** vasopressor dependency index (mmHg^−1^); **d** arterial lactate (mmol/L); **e** fluid balance (mL) CSS and HTx. Data are expressed as mean ± SEM, *n* = 4/group. Top *F* and *p* values on each graph represent effect of time, and bottom *F* and *p* values represent differences between BSD vs. sham over time. *B* Baseline, *ST* stabilisation 1 h following completion of instrumentation procedures in recipient, CPB—establishment of cardiopulmonary bypass, 0H—successful weaning from CPB

Following successful weaning from CPB, MAP (Fig. 5a) and HR (Fig. 5b) remained stable post-HTx through increasing vasoactive support, regardless of donor heart group (Fig. 5c; see Additional file 1: Fig S6 for weight-based doses of adrenaline, noradrenaline, vasopressin and dopamine individually). Arterial lactate consistently increased post-HTx, with no differences observed between groups (Fig. 5d; Sham: 7.6 ± 2.8; BSD: 11.3 ± 2.5 mmol/L). Sham recipients exhibited a slightly greater fluid balance early post-HTx (Fig. 5e), however this was no different 6 h post-HTx. Hemodynamic and ventilatory parameters, blood results and vasoactive support during the course of the study at baseline, T0, T3 and T6 in recipient animals are available in Additional file 1: Table S3.

**Fig. 6:**
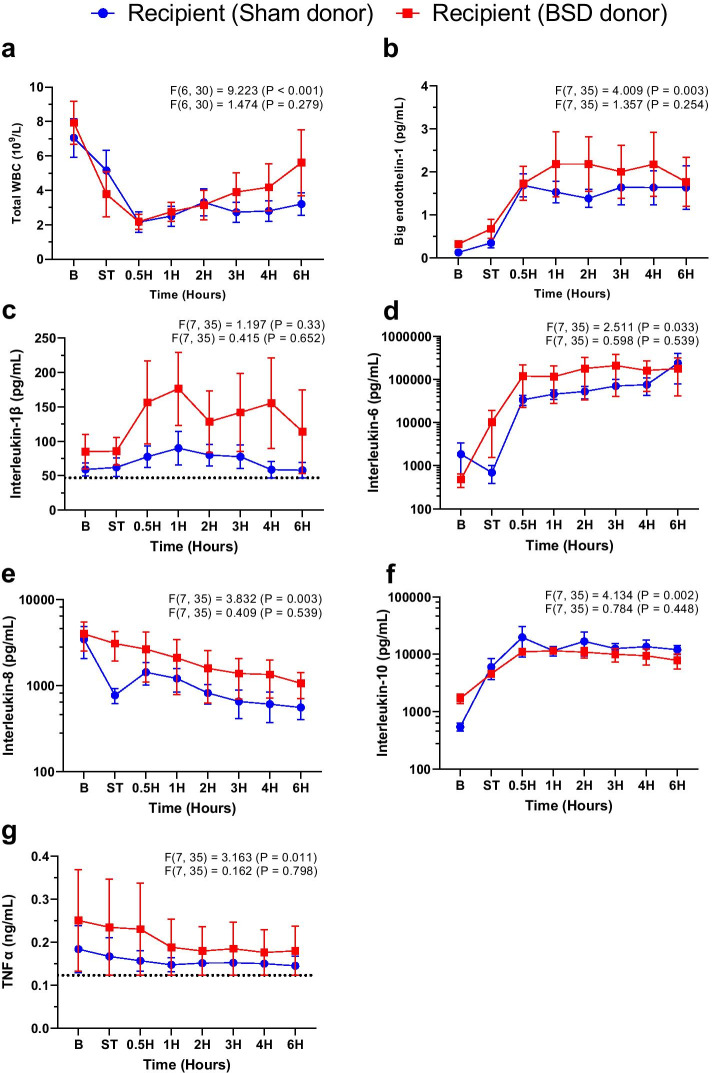
Systemic inflammation in recipients post-HTx. Changes in plasma **a** total white blood cells (WBC, 10^9^/L); **b** big endothelin-1 (BET-1, pg/mL); **c** interleukin 1β (pg/mL); **d** interleukin-6 (pg/mL); **e** interleukin-8 (pg/mL); **f** interleukin-10 (pg/mL); and **g** TNFα (ng/mL) following confirmation of donor BSD (or sham). Data are expressed as mean ± SEM, *n* = 4/group. Top *F* and *p* values on each graph represent effect of time, and bottom *F* and *p* values represent differences between BSD vs. sham over time. *B* Baseline, *ST* stabilisation 1 h following completion of instrumentation procedures in recipient, 0H—successful weaning from CPB. Dotted lines on **c** and **g** represent the lowest detectable limit of the respective cytokine ELISA

No significant differences in total white blood cell count, BET-1 or systemic cytokine expression (IL-1β, IL-6, IL-8, IL-10, TNFα) were observed between groups (Fig. 6). BET-1, IL-6, IL-10 increased significantly post-HTx in both groups, while WBC, IL-8 and TNFα decreased.

### Pre- and post-HTx cardiac function

**Fig. 7:**
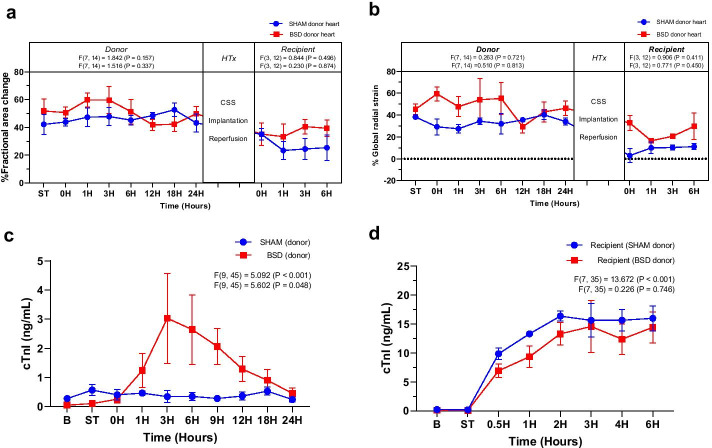
Cardiac function in donors (pre-HTx) and recipients (post-HTx). Cardiac function as determined by changes in **a** % fractional area change and **b** % global radial strain by two-dimensional speckle tracking echocardiography. Cardiac damage was assessed by cardiac troponin I (cTnI, ng/mL) levels in plasma of **c** donors and **d** recipients. Data are expressed as mean ± SEM, *n* = 4/group. Top *F* and *p* values on each graph represent effect of time, and bottom *F* and p values represent differences between BSD vs. sham over time. *B* Baseline, *ST* stabilisation 1 h following completion of instrumentation procedures, 0H in donor—confirmation of BSD, 0H in recipient—successful weaning from CPB

Epicardial echocardiography revealed no significant differences in ventricular systolic function between sham and BSD donor hearts (Fig. 7a and b) prior to heart procurement. Following BSD onset, plasma cTnI levels were significantly elevated compared to sham (Fig. 7c), slowly decreasing and no different at 24 h. Following CSS and HTx, ventricular systolic function was slightly reduced compared to pre-HTx function (Fig. 7a and b), but no significant differences were detected between groups. Similarly, post-HTx cTnI levels increased in the initial 2 h following successful weaning from CPB, but no differences were detected between groups (Fig. 7d).

## Discussion

This is a novel sheep HTx model incorporating 24 h donor BSD, with recipients successfully weaned from CPB and monitored for 6 h post-HTx. In comparison to previous models (pigs and dogs) [13], the clinical value of the presented HTx model is summarised below:

1. Animal selection: Sheep were chosen for their similarities in size/weight [27], respiratory, hemodynamic, and microcirculatory physiology with humans, that are critical to HTx [28].
2. Twenty-four hours BSD: Our model is representative of common timeframes of clinical declaration of BSD. Few studies have published HTx incorporating BSD beyond 6 h in large animals [12, 29].
3. CSS: The cold and total ischemic times utilised match clinical boundaries deemed safe for recipients [5], and sufficiently induced significant cardiac damage in sheep hearts that appear vulnerable to CSS-mediated injury.
4. HTx technique: An orthotopic bicaval HTx technique was utilised to maintain consistency with contemporary HTx surgery [15].
5. Post-HTx cardiac function: All animals were weaned from CPB despite the clinical and technical challenges of this model. Echocardiography permitted non-invasive assessment of cardiac function commonly used in ICU post-HTx. Similar animal models are largely reliant upon invasive P–V analyses.

### BSD-mediated hemodynamics and inflammation

Brain death occurs following significant and persistent elevations in ICP, leading to exaggerated hemodynamic perturbations, commonly requiring vasoactive support. Despite donor management and optimisation strategies, cardiac dysfunction occurs in BSD due to systemic catecholamine response and impaired cardiac contraction [13, 30], which can impact acceptance of a donor heart. For example, in Australia, ~ 70% of donor hearts not retrieved for HTx are declined due to either being ‘not medically suitable’ (48%) or age of the donor heart (22%) [31]. Excessive BSD-mediated cardiac dysfunction [4] and older donor age [5] significantly increase the probability of PGD after HTx. In our model, significant catecholamine overload impaired hemodynamic control during BSD (e.g., tachycardia, elevated MAP) and required vasopressor management. This early hemodynamic compromise was coupled with systemic IL-6 and IL-8 elevations, which resolved and was comparable to sham 24 h later. Exaggerated systemic inflammation is a hallmark feature of BSD [30, 32], well-reported in animals [33–35] and humans [36–38]. Increased IL-6 levels occurs systemically [39, 40], and in heart [41] and brain [40] tissue with BSD/brain injury, albeit on different time courses. Elevated plasma IL-6 during BSD correlates with reduced hospital-free survival six months post-transplant and organ yield [42]. The observed transient rise in plasma IL-8 here is consistent with clinical observations [39], and the severity of cardiac allograft rejection increases with elevated neutrophil infiltration [43]. No changes in plasma proinflammatory TNFα and IL-1β were evident in our study, which aligns with other reports [39, 44, 45]. Our observation of increased plasma IL-10 reflects observations in BSD patients [42, 46, 47]. However, the delay in activation of IL-10 immunosuppressive actions (> 8 h) is unlikely responsible the unchanged TNFα and IL-1β in our study [48]. Plasma BET-1 peaked early and further delineates BET-1 patterns during BSD following our previous reports [14]. Both ET-1 [49, 50] and BET-1 [51–53] may improve risk stratification of acute myocardial infarction and heart failure, and help predict outcomes post-HTx [54]. Despite the observed BSD-mediated pathophysiological changes, all BSD donor hearts were transplantable and successfully weaned from CPB post-HTx. Thus, our model accurately mimics the clinical scenario of transplantable BSD hearts exposed to characteristic hemodynamic and inflammatory perturbations.

### Heart preservation and transplantation

Donor heart preservation methods are unchanged for over 40 years [7]. In this model, hearts were procured after 24 h monitoring, preserved by CSS and then transplanted. Significant heterogeneity and variation in reporting exists in published animal models of BSD-HTx regarding heart preservation [13]. Ischemic time duration during donor heart preservation is an independent risk factor for short- and long-term mortality post-HTx [5]. Indeed, the cardiac graft quality is directly proportional to the cold ischemic time [55], and the PGD risk greatly increases following heart preservation beyond 4 h. The median cardiac allograft ischemic time is 3.2 (1.5–5.0) hours (2009–2016) [5]. Comparatively, we used a total ischemic time slightly less than the reported median [5], but certainly still within the clinically relevant range. Sheep are commonly used as reliable and reproducible preclinical models of chronic heart failure and myocardial infarction, despite an accepted 30% mortality rate, predominantly due to malignant arrhythmias [56–58]. Sheep and humans have similarities in cardiac myocyte, electrophysiological, contractile and metabolic properties [56, 59]. The heart is similarly exposed to significant ischemia during HTx, and we commonly observed intractable ventricular fibrillation upon reperfusion, often requiring repeated electrical defibrillation and pharmacological resuscitation. Regardless, we successfully restarted and weaned all hearts from CPB post-HTx.

### Post-HTx status and cardiac function

After weaning from CPB, recipient hemodynamic function was stable, but required increasing vasoactive support and was associated with increasing blood lactate. Our observations of increasing vasoactive requirements with concomitant elevations in blood lactate in the early post-operative period mimic clinical HTx observations [60–62]. Post-HTx hyperlactatemia appears common, generally resolves without significant adverse outcomes [60–62]. Based on clinical observations [60, 62], peak lactate levels post-HTx in our study may not have been reached, and may have been influenced by allograft ischemic time [61, 62] and vasoactive use [60, 62]. While cardiac function was depressed post-HTx, relative to donor baseline function, it appeared stable, and cTnI release plateaued early post-HTx. Comparative analysis with other BSD-HTx animal models is difficult as few studies [29, 63] have utilised echocardiography to assess cardiac function, with invasive pressure–volume (P–V) relationship analyses preferred [13]. Speckle-tracking echocardiography to determine longitudinal strain can be supplementary to invasive composite contractility index derived from P–V relationship analyses [64]. Furthermore, traditional echocardiography measures (e.g., ejection fraction and fractional shortening) are less representative of cardiac contractility, but significantly associated with left ventricular load and mass [64]. Schroeder et al. measured pediatric cardiac dysfunction post-HTx, and found that finding no single clinical variable was significantly associated with P–V relationship analyses [65]. These disparities in cardiac function assessment render comparison across studies difficult.

Interestingly, despite the profound hemodynamic and inflammatory perturbations in BSD donors, no major differences were evident in post-HTx hemodynamic, inflammatory or cardiac function parameters between groups. Our ability to successfully wean recipients from CPB following 24 h BSD is likely associated with reduced systemic inflammation at time of heart retrieval. During preliminary BSD-HTx studies, hearts exposed to 6 h BSD could not be weaned from CPB. During these studies, the cold ischemic time was comparable to the current study, and only differed in the length of or complete exposure to donor BSD. Other HTx animal studies incorporate BSD exposure up to 6 h [13], which is not reflective of clinical practice [66, 67]. In Australia (2014–2018), the median time from confirmation of brain death to donation was 21.1–22.7 h [68], in line with the model presented here. Clinical outcomes post-HTx are inconsistent regarding the influence of extended BSD donor management (longer exposure to BSD injury) upon recipient survival. Some reports show that extended donor management improves rejection-free survival, with no effect upon mortality in pediatric patients (4–17 days donor management) [69], or adult recipient survival post-HTx (~ 19 h donor management) [67]. Conversely, others report that extended donor management > 14 h [70] or > 72 h [71] is linked to poorer recipient survival. Furthermore, extending donor management time to optimise cardiac function may significantly increase the number of hearts transplanted. Borbely et al. demonstrated that 52% of donor hearts exhibiting cardiac dysfunction at initial assessment via transthoracic echocardiography (TTE) could be transplanted by extending donor management and performing serial TTE [72], which may be reflective of our observations.

### Strengths and limitations

Developing large animal models that accurately represent clinical scenarios is complex, resource-intensive, expensive and often requires similar hospital technology and facilities. Development of this model was a complex and labour-intensive process, but was strengthened by the comprehensive research and clinical team with extensive experience in clinical and animal experimentation in critical care medicine and HTx. We used advanced ICU monitoring systems and protocols to increase the potential for clinically translatable outcomes. Nevertheless, the current study has several limitations. Despite all care taken to assess animal health prior to experimentation, and use of prophylactic broad-spectrum antibiotics throughout the study, there is potential, though unlikely, that concomitant undiagnosed infections could have contributed to the catecholamine storm in donors. This study has a small sample size, and thus meaningful statistical analysis is challenging. However, we have comprehensively assessed all donors and recipients over the entire time course of the experimental procedure. A longer post-HTx monitoring period would have been beneficial, but better suited to a smaller interventional study, where assessment of long-term outcomes is critical to efficacy of a desired intervention for patient survival.

## Conclusions

Cardiac transplantation is a complex field, where recipient outcomes are dependent upon an extended timeline that begins with donor injury. Any model hoping to advance the HTx field must incorporate the explosive brain insult, ICU care and hormone resuscitation, donor heart preservation, reimplantation and weaning from CPB. This model represents the first ovine model of bicaval HTx incorporating donor BSD for 24 h. Donor BSD induced significant hemodynamic and inflammatory perturbations requiring vasoactive management, yet was comparable to sham donors after 24 h. Following CSS and HTx, all animals were successfully weaned from CPB and monitored for up to 6 h (except one), with no significant differences in post-HTx hemodynamic, inflammatory or cardiac function between groups. This ovine model of BSD-HTx may have a promising role in evaluating novel peri-transplant techniques and therapies, with the aim of ultimately improving both the quality and quantity of donor hearts.

## Supplementary Information

**Additional file 1.** **Table S1.** Consumable details relevant to the methodology. **Table S2.** Hemodynamic and ventilatory parameters, blood results and vasoactive use during the course of the study at stabilisation (one hour following completion of all instrumentation procedures), T0 (confirmation of BSD/Sham), T12 and T24 in donor animals. All values are mean (SD). NA – not available; FiO_2_ – inspiratory fraction of oxygen; PEEP – positive end-expiratory pressure; EtCO_2_ – end-tidal carbon dioxide; PaO^2^ – arterial partial pressure of oxygen; PaCO^2^ – arterial partial pressure of carbon dioxide; HCO_3_ – concentration ofbicarbonate in arterial blood. **Table S3.** Hemodynamic and ventilatory parameters, blood results and vasoactive use during the course of the study at stabilisation (one hour following completion of all instrumentation procedures), T0 (successful weaning from CPB), T1, T3 and T6 in recipient animals. All values are mean (SD). NA – not available; FiO_2_ – inspiratory fraction of oxygen; PEEP – positive end-expiratory pressure; EtCO_2_ – end-tidal carbon dioxide; PaO^2^ – arterial partial pressure of oxygen; PaCO^2^ – arterial partial pressure of carbon dioxide; HCO_3_ – concentration of bicarbonate in arterial blood. **Table S4.** P values from multiple comparisons test for parameter estimates among Sham and BSD donors at each time point for donor heart rate (HR), plasma metanephrines (Met.), mean arterial pressure (MAP), vasopressor dependency index (VDI), blood lactate, minute volume and fluid balance. B – baseline, ST – stabilisation, T0 – confirmation of BSD, T1-T24 – 1-24 hours post-BSD confirmation.

## Data Availability

All data generated or analysed during this study are included in this published article (and its Additional Information files).

## Abbreviations

ACT: Activated clotting time
AoP: Aortic pressure
BET-1: Big endothelin-1
BSA: Body surface area
BSD: Brainstem death
CPB: Cardiopulmonary bypass
CSS: Cold static storage
cTnI: Cardiac troponin I
CVP: Central venous pressure
ECG: Electrocardiogram
ETCO_2_: End-tidal carbon dioxide
FAC: Fractional area change
FiO_2_: Inspiratory fraction of oxygen
GRS: Global radial strain
HCO_3_: Concentration of bicarbonate in arterial blood
HR: Heart rate
HTx: Heart transplantation
ICP: Intracranial pressure
IL: Interleukin
IRI: Ischemia–reperfusion injury
LVAD: Left ventricular assist device
MAP: Mean arterial pressure
PaCO_2_: Arterial partial pressure of carbon dioxide
PaO_2_: Arterial partial pressure of oxygen
PAP: Pulmonary artery pressure
PEEP: Positive end-expiratory pressure
PGD: Primary graft dysfunction
PSAX: Parasternal short axis
P–V: Pressure–volume
RR: Respiratory rate
SpO_2_: Oxygen saturation by pulse oximetry
TNFα: Tumour necrosis factor alpha
TTE: Transthoracic echocardiography
VDI: Vasopressor dependency index
WBC: White blood cells
