# Supplementary material for: A clinically relevant sheep model of orthotopic heart transplantation 24 h after donor brainstem death
Source: Intensive Care Med Exp. 2021 Dec 24;9:60. doi: 10.1186/s40635-021-00425-4 (PMC8702587; doi:10.1186/s40635-021-00425-4)
Supplement: Supplementary file 1 — Additional file 1. Table S1. Consumable details relevant to the methodology. Table S2. Hemodynamic and ventilatory parameters, blood results and vasoactive use during the course of the study at stabilisation (one hour following completion of all instrumentation procedures), T0 (confirmation of BSD/Sham), T12 and T24 in donor animals. All values are mean (SD). NA – not available; FiO2 – inspiratory fraction of oxygen; PEEP – positive end-expiratory pressure; EtCO2 – end-tidal carbon dioxide; PaO2 – arterial partial pressure of oxygen; PaCO2 – arterial partial pressure of carbon dioxide; HCO3 – concentration ofbicarbonate in arterial blood. Table S3. Hemodynamic and ventilatory parameters, blood results and vasoactive use during the course of the study at stabilisation (one hour following completion of all instrumentation procedures), T0 (successful weaning from CPB), T1, T3 and T6 in recipient animals. All values are mean (SD). NA – not available; FiO2 – inspiratory fraction of oxygen; PEEP – positive end-expiratory pressure; EtCO2 – end-tidal carbon dioxide; PaO2 – arterial partial pressure of oxygen; PaCO2 – arterial partial pressure of carbon dioxide; HCO3 – concentration of bicarbonate in arterial blood. Table S4. P values from multiple comparisons test for parameter estimates among Sham and BSD donors at each time point for donor heart rate (HR), plasma metanephrines (Met.), mean arterial pressure (MAP), vasopressor dependency index (VDI), blood lactate, minute volume and fluid balance. B – baseline, ST – stabilisation, T0 – confirmation of BSD, T1-T24 – 1-24 hours post-BSD confirmation. [file 40635_2021_425_MOESM1_ESM.docx]

**SUPPLEMENTAL DIGITAL CONTENT**

**Title:** A clinically relevant sheep model of orthotopic heart transplantation following 24 hours donor brain death.

**Author list:** Louise E See Hoe^1,2^*, Karin Wildi^1,2,3^*, Nchafatso Obonyo^1,2,4,5^, Nicole Bartnikowski^1,6^, Charles McDonald^1,7^, Kei Sato^1,2^, Silver Heinsar^1,2,8^, Sanne Engkilde-Pedersen^1,9^, Sara Diab^1,2^, Margaret Passmore^1,2^, Matthew Wells^1,10^, Ai Chi Boon^1,2^, Arlanna Esguerra^1,9^, David G Platts^1,2^, Lynnette James^11^, Mahe Bouquet^1,2^, Kieran Hyslop^1,2^, Tristan Shuker^1,12^, Carmen Ainola^1,2^, Sebastiano Maria Colombo^1,2,13^, Emily S Wilson^1,2^, Jonathan E Millar^1,2,14^, Maximillian Malfertheiner^1,15^, Janice D Reid^1,2,12^, Hollier O'Neill^1,2^, Samantha Livingstone^1,2^, Gabriella Abbate^1,2^, Noriko Sato^1,2^, Ting He^11^, Viktor von Bahr^16^, Sacha Rozencwajg^1,17^, Liam Byrne^1,18,19^, Leticia Pretti Pimenta^1^, Lachlan Marshall^1,11,20^, Lawrie Nair^1,20^, John-Paul Tung^1,2,9,21^, Jonathan Chan^20,22^, Haris Haqqani^2,20^, Peter Molenaar^2,23^, Gianluigi Li Bassi^1,2,24^ Jacky Suen^1,2,12^ David C McGiffin^25,26^, John F Fraser^1,2^.

* - co-first author

**Corresponding author**: Dr Louise E See Hoe ([l.seehoe@uq.edu.au](mailto:l.seehoe@uq.edu.au))

Critical Care Research Group, The Prince Charles Hospital, Queensland, Australia; Prince Charles Hospital Northside Clinical Unit, Faculty of Medicine, University of Queensland, Queensland, Australia

Business address: Room 13, Level 3, Clinical Sciences building, The Prince Charles Hospital

627 Rode Rd, Chermside, Queensland, 4032, AUSTRALIA

**Table S1:** Consumable details relevant to the methodology

| **Reference section in manuscript** | **Purpose** | **Item details** | **Company** |
| --- | --- | --- | --- |
| ***Animal preparation (both donors and recipients)*** | **External jugular vein cannulation** | 1. 8.5 Fr central venous line 2. 8 Fr venous sheath | 1. Arrow Int., Reading USA 2. Edwards Lifesciences, Irvine, USA |
|  | **Intubation** | 8-10 internal diameter endotracheal tube | Lo-Pro, Covidien, USA |
|  | **Suction of gastric secretions** | 14 Fr nasogastric tube | Covidien, USA |
|  | **Suction of oropharyngeal secretions** | Yankauer suction handle | ConvaTec, United Kingdom |
|  | **Femoral artery cannulation** | Cook Femoral Artery Nylon Pressure Monitoring Catheter Set | Cook Medical, USA |
|  | **Continuous cardiac output monitoring** | 7.5 Fr Swan-Ganz CCombo pulmonary artery catheter | Edwards Lifesciences, USA |
|  | **Urine output monitoring and sampling** | 12 Fr Foley urinary catheter | Bard Limited, USA |
| ***Donor-specific preparation and critical care management*** | **Coronary sinus blood sampling** | 7 Fr 3-lumen central venous catheter | CVL; Arrow Int., Reading USA |
|  | **Donor heart preservation** | St Thomas’s cardioplegic solution (Cat# AHB7832 Cardioplegia A solution) | Baxter Healthcare, Australia |
| ***Recipient-specific preparation, orthotopic heart transplantation, and critical care management*** | **CPB circuit oxygenation** | Capiox FX Oxygenator, Cat# CX*FX15RE30 | Terumo, Australia |
|  | **Aortic cannulation in recipient for HTx** | 16-Fr elongated one-piece EOPA^®^ cannula | Medtronic, USA |
|  | **Bicaval cannulation in recipient for HTx** | 24-Fr and 28-Fr right-angled cannulae | Medtronic, USA |
|  | **Continuous cardiac output monitoring** | 7.5 Fr Swan-Ganz CCombo pulmonary artery catheter | Edwards Lifesciences, USA |

**Figure S1:** A mini left thoracotomy was performed through the upper border of the 5th costal bone to cannulate the azygos vein and gain access to the coronary sinus for blood sampling. Epicardial echocardiography was also performed in the donor through the thoracotomy window.


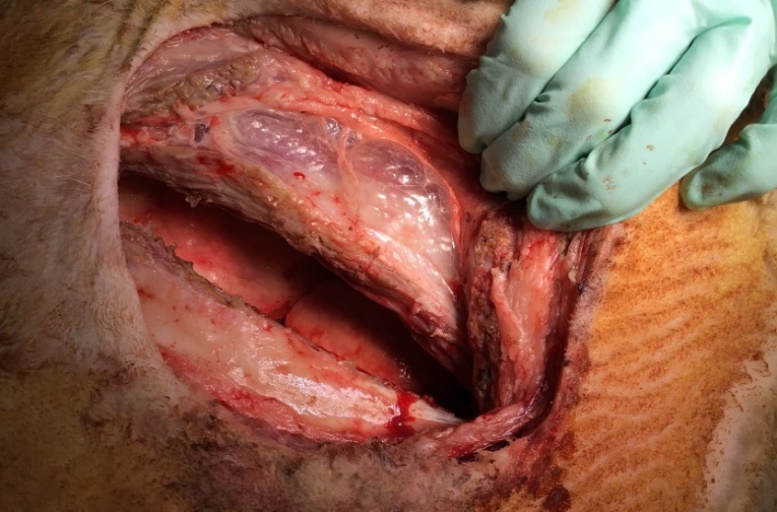


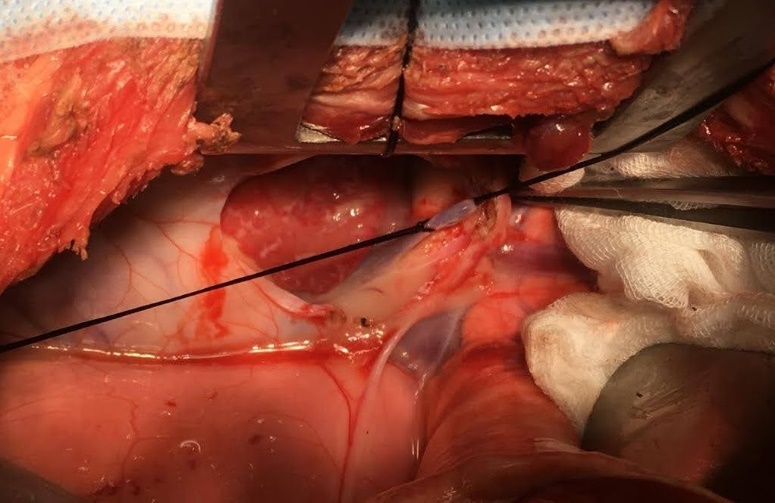


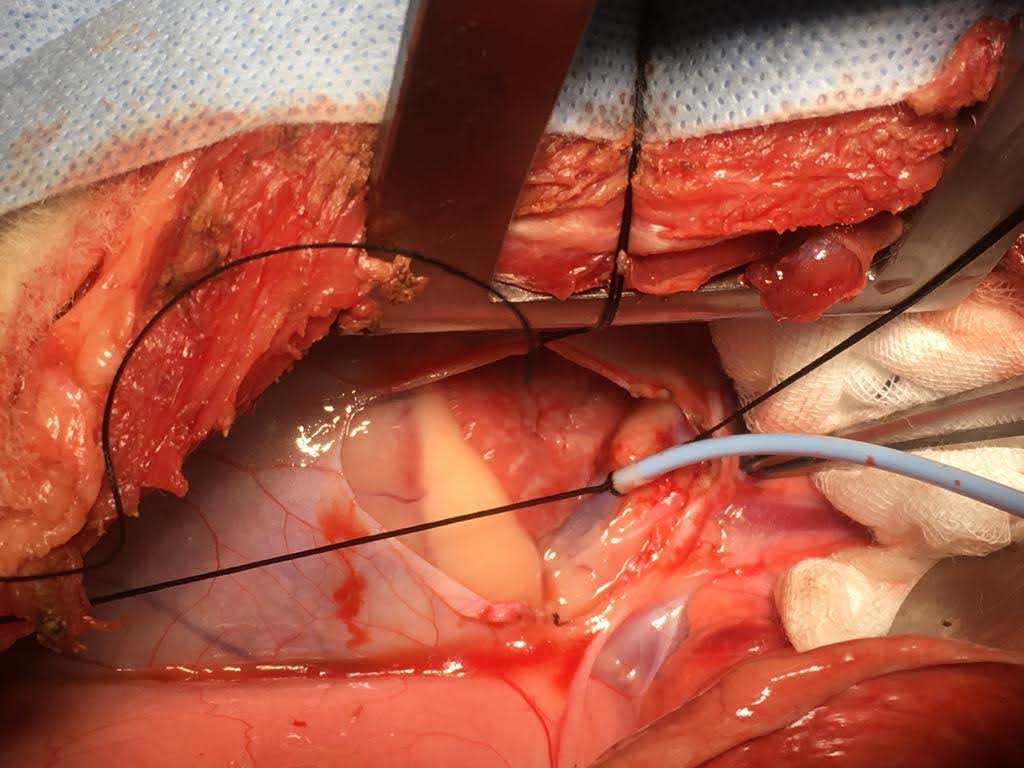


**Figure S2:** A midline incision on the skull was used to expose sagittal and lambdoid sutures and create burr holes required for BSD induction and an intracranial pressure monitor.


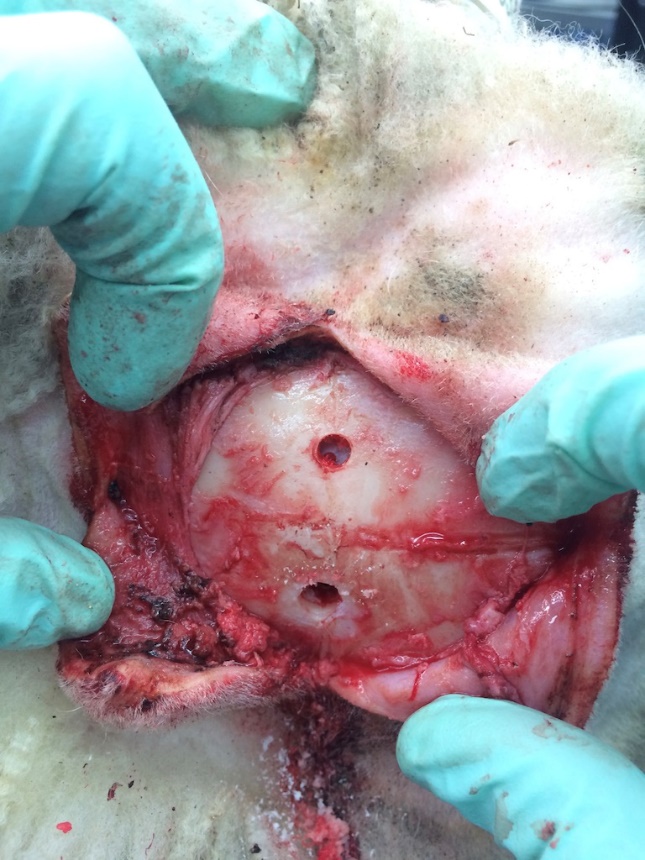


**Figure S3:** After 24 hours monitoring in the donor, the animal was placed supine position and a sternotomy was performed to explant the heart. The ascending aorta in sheep is very short prior to dividing into the innominate artery and aortic arch. A purse-string suture was placed in the ascending aorta for the cardioplegic needle.


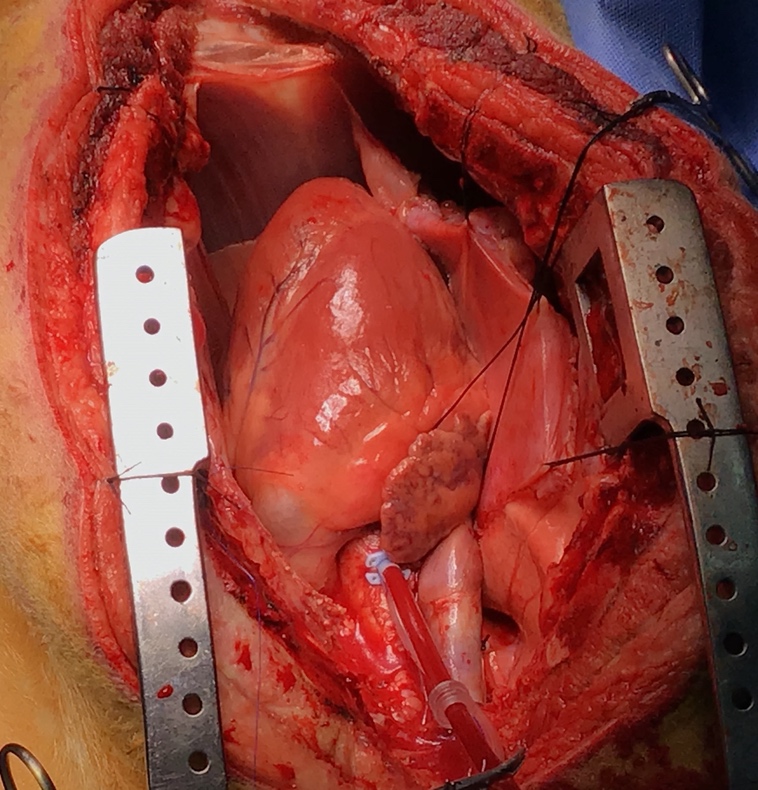


**Figure S4.** Example experimental intensive care settings. Both the donor and recipient are monitored in intensive care settings. This picture depicts the donor coming to completion of 24 hours BSD, awaiting heart retrieval, and the recipient following completion of instrumentation procedures prior to establishment of cardiopulmonary bypass. At this point in the experiment, both animals are monitored at the same time.


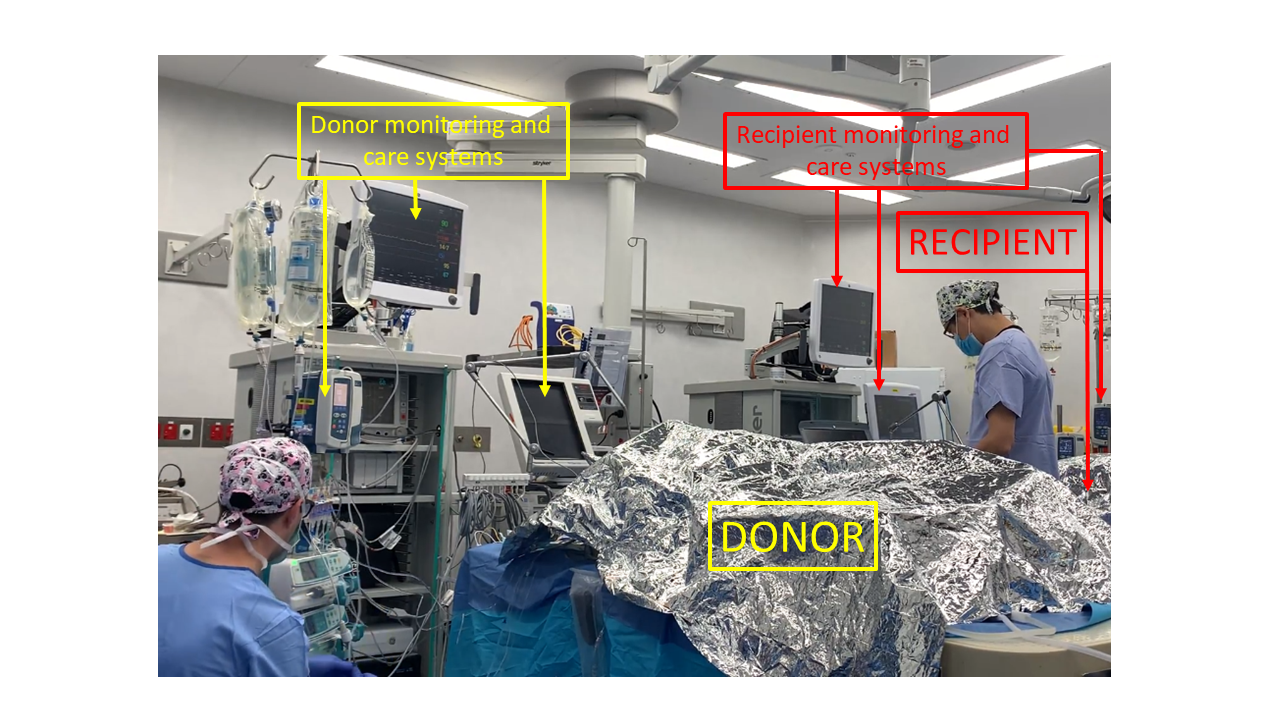


**Figure S5.** Example experimental surgical settings. This image depicts the typical surgical settings used for these experiments. Here, the recipient animal is being prepared and the chest opened, while the cardiopulmonary bypass system is being prepared for surgery. The donor heart has been retrieved, and the donor animal removed from the surgical theatre.


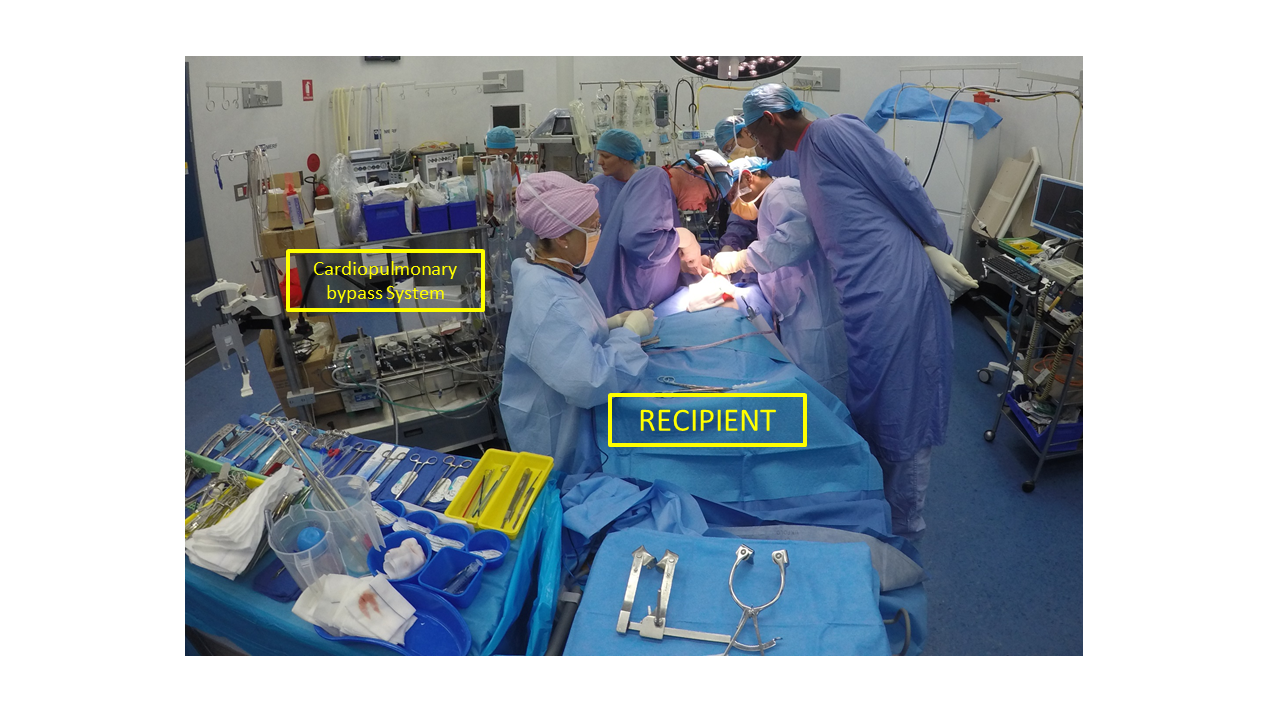


***Orthotopic HTx procedures:***

All recipients were prepared and instrumented as stated in ‘Animal preparation’. Recipient animals were then rested for 1 hour to achieve vital signs stability. Standard experimental settings for monitoring both donor and recipient are depicted in Fig S4, and standard surgical settings are depicted in Fig S5. Following administration of vecuronium (0.2 mg/kg i.v.), the recipient was placed in supine position and the chest opened via median sternotomy. Purse-string sutures were placed in the ascending aorta, level with the take-off of the aortic arch, superior vena cava and inferior vena cava extra-pericardially within the pleural space. Caval tapes were placed around the superior and inferior vena cava. The CPB circuit (Capiox FX Oxygenator, Cat# CX*FX15RE30, Terumo, Australia) was primed with 2-3 units of compatible ovine packed red blood cells (1). Heparin (100-300 U/kg, to achieve ACT >400 sec) was administered intravenously. Cardiopulmonary bypass was established with a single aortic cannula (16-Fr elongated one-piece EOPA^®^ cannula, Medtronic, USA) and bicaval cannulation (24-Fr and 28-Fr right-angled cannulae, Medtronic, USA), and perfusate temperature reduced to a core temperature of 32°C. Fluid infusions were stopped upon CPB commencement. The azygos vein was ligated in the left pleural space. The ascending aorta was cross-clamped and the recipient heart was removed by transecting the ascending aorta, main pulmonary artery, and superior vena cava at the cavoatrial junction, dividing the inferior vena cava at the right atrial inferior vena cava junction. The heart was then transected to form the left atrial cuff through the AV groove, for the specific purpose of providing thicker muscle in the left atrial cuff and ensure that the left atrial suture line was quite hemostatic.

Once CPB was established and the recipient heart removed, standard orthotopic HTx was performed. The recipient left atrial cuff circumference formed by the AV groove tissue was smaller than if the cuff was fashioned by taking the excision line through the thin left atrial wall. Therefore, the left donor pulmonary veins were oversewn (5/0 Prolene) since incising the muscle between all of the pulmonary veins would have resulted in an excessively large donor left atrial cuff. The right sided pulmonary vein orifices only needed a small amount of enlargement to match the recipient left atrial cuff. Anastomoses were performed in the following order: left atrium (continuous 4/0 Prolene), inferior vena cava (continuous 4/0 Prolene), superior vena cava (continuous 5/0 Prolene), pulmonary artery (continuous 5/0 Prolene), ascending aorta (continuous 4/0 Prolene). After completion of the pulmonary artery anastomosis, amiodarone (150 mg), lignocaine (50 mg), MgSO_4_ (10 mmoL) and methylprednisolone (250 mg) were administered intravenously, and a 7.5 Fr pulmonary artery catheter (Swan-Ganz CCombo, Edwards Lifesciences, USA) was inserted through the right jugular introducer for continuous cardiac output monitoring (prior to completion of aortic anastomoses). Fluid infusions recommenced once the animal was rewarmed to 37.0°C. Following completion of all anastomoses, de-airing was performed with a needle vent in the ascending aorta and the aortic cross-clamp removed.

References:

1. Simonova G, Tung JP, Fraser JF, Do HL, Staib A, Chew MS, Dunster KR, Glenister KM, Jackson DE, Fung YL (2014) A comprehensive ovine model of blood transfusion. Vox Sang 106:153-160 doi:10.1111/vox.12076

**Figure S6.** Weight-based doses of drugs used to provide hemodynamic support post-HTx in recipients. Recipient animals received critical care management for an additional 6 hours following successful separation from CPB, and hemodynamics were adjusted to maintain mean arterial pressure (MAP) > 65 mmHg with a) noradrenaline (µg/kg/min), b) adrenaline (µg/kg/min), c) dopamine (µg/kg/min) and d) vasopressin (IU/kg/min). All data are presented as mean ± SEM.

**
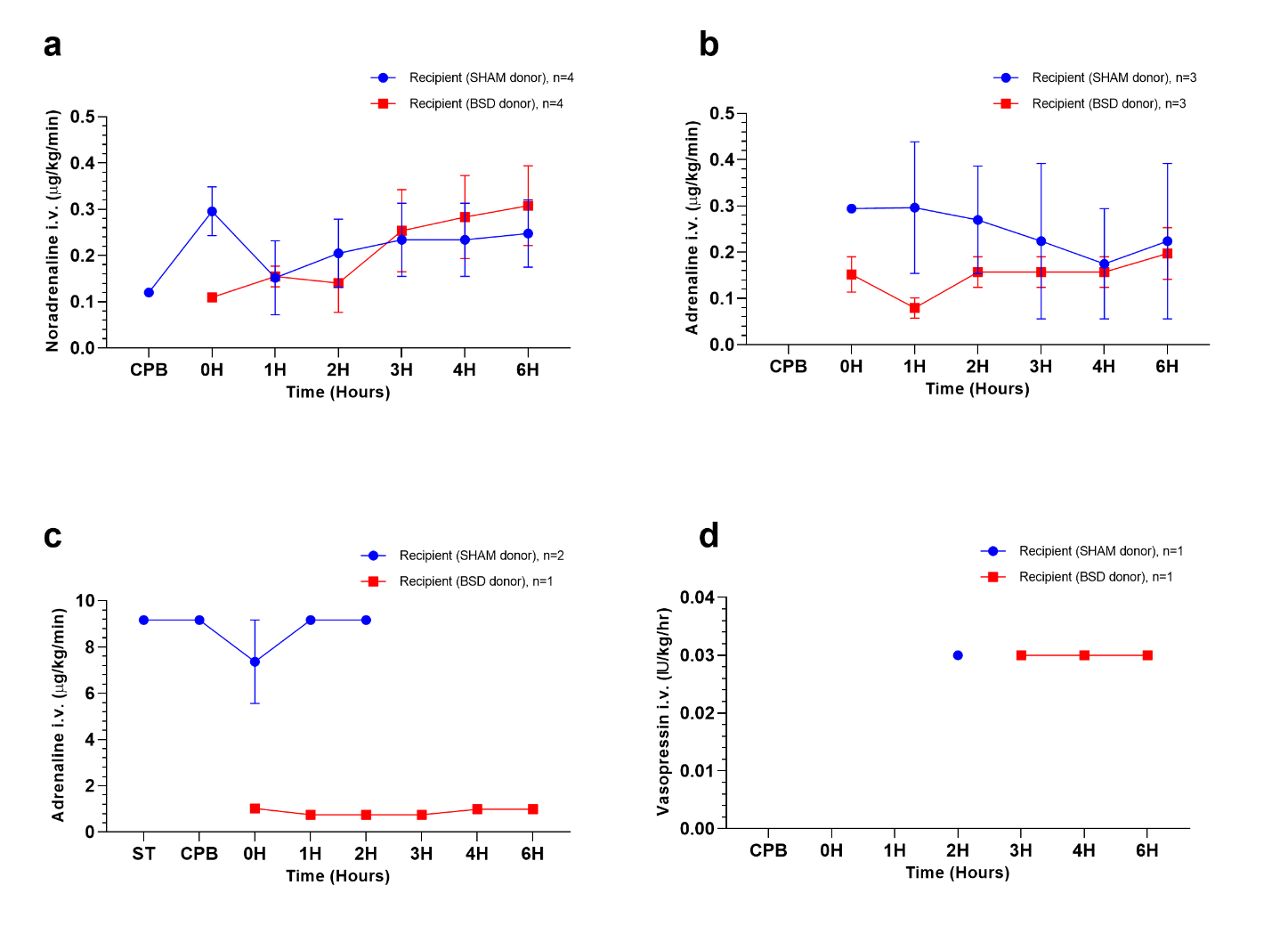
**

**Table S2.** Hemodynamic and ventilatory parameters, blood results and vasoactive use during the course of the study at stabilisation (one hour following completion of all instrumentation procedures), T0 (confirmation of BSD/Sham), T12 and T24 in donor animals. All values are mean (SD). NA – not available; FiO_2_ – inspiratory fraction of oxygen; PEEP – positive end-expiratory pressure; EtCO_2_ – end-tidal carbon dioxide; PaO^2^ – arterial partial pressure of oxygen; PaCO^2^ – arterial partial pressure of carbon dioxide; HCO_3_ – concentration of bicarbonate in arterial blood.

|  | *Stabilisation* | | *0 hr* | | *12 hr* | | *24 hr* | |
| --- | --- | --- | --- | --- | --- | --- | --- | --- |
|  | **SHAM** | **BSD** | **SHAM** | **BSD** | **SHAM** | **BSD** | **SHAM** | **BSD** |
| *Hemodynamics* |  |  |  |  |  |  |  |  |
| Heart rate (bpm) | 113 (34) | 108 (23) | 118 (45) | 158 (35) | 108 (19) | 102 (27) | 97 (35) | 86 (17) |
| Mean arterial pressure (mmHg) | 106 (6) | 85 (17) | 101 (4) | 57 (9) | 85 (7) | 79 (11) | 95 (24) | 68 (9) |
| Central venous pressure (mmHg) | 10 (5) | 9 (5) | 9 (5) | 10 (5) | 9 (4) | 9 (5) | 13 (4) | 12 (3) |
| Vasopressor dependency index (mmHg^-1^) | 0 (0) | 0.01 (0.02) | 0 (0) | 0.20 (0.14) | 0.02 (0.05) | 0.05 (0.05) | 0.04 (0.09) | 0.06 (0.12) |
| Systemic vascular resistance index (dynes – sec/cm^-5^/m^2^ | 1174 (1015) | NA | 1146 (991) | 407 (249) | 700 (493) | 706 (164) | 532 (372) | 692 (136) |
| Calculated cardiac output (L/min) | 3.93 (0.15) | 6.25 (3.04) | 3.97 (0.38) | 7.97 (2.63) | 5.50 (1.21) | 5.55 (1.14) | 6.40 (2.28) | 4.38 (1.09) |
| Intracranial pressure (mmHg) | 36 (10) | 90 (135) | 36 (3) | 280 (41) | 81 (80) | 156 (148) | NA | 300 (NA) |
| Body temperature (°C) | 37.7 (0.7) | 37.8 (0.9) | 37.6 (0.9) | 37.4 (1.0) | 39.7 (0.4) | 37.7 (2.1) | 39.0 (1.6) | 37.6 (1.7) |
| *Vasoactive use (μg/kg/min)* |  |  |  |  |  |  |  |  |
| Noradrenaline | 0 (0) | 0.004 (0.08) | 0 (0) | 0.10 (0.07) | 0.02 (0.04) | 0.04 (0.05) | 0.03 (0.06) | 0.04 (0.07) |
| Dopamine | 0 (0) | 0 (0) | 0 (0) | 1.69 (1.18) | 0 (0) | 0 (0) | 0 (0) | 0 (0) |
| *Ventilation* |  |  |  |  |  |  |  |  |
| FiO_2_ (%) | 38 (3) | 40 (0) | 38 (3) | 43 (4) | 38 (3) | 40 (4) | 36 (3) | 41 (6) |
| Minute volume (L/min) | 5.1 (0.5) | 6.4 (0.54) | 5.1 (0.5) | 7.8 (1.86) | 6.2 (1.3) | 11.0 (0.74) | 6.6 (2.8) | 8.6 (1.41) |
| PEEP (cm H_2_O) | 10.0 (0) | 7.8 (2.1) | 9.5 (1) | 7.8 (1.9) | 9 .0 (1.2) | 8.3 (2.4) | 9.0 (1.2) | 7.8 (2.1) |
| EtCO_2_ (mmHg) | 36.0 (3.2) | 38.3 (4.3) | 37.3 (2.2) | 48.5 (9.0) | 43.8 (3.2) | 34.8 (8.9) | 38.0 (6.3) | 37.5 (4.7) |
| *Arterial Blood gases* |  |  |  |  |  |  |  |  |
| pH | 7.47 (0.04) | 7.43 (0.11) | 7.41 (0.07) | 7.30 (0.12) | 7.35 (0.12) | 7.46 (0.03) | 7.36 (0.05) | 7.42 (0.04) |
| PaO_2_ (mmHg) | 137 (30) | 100 (29) | 122 (19) | 120 (35) | 111 (10) | 124 (26) | 93 (13) | 123 (28) |
| PaCO_2_ (mmHg) | 34 (4) | 38 (6) | 41 (7) | 53 (11) | 48 (17) | 38 (6) | 44 (6) | 41 (2) |
| Lactate (mmol/L) | 1.0 (0.25) | 1.97 (2.22) | 1.33 (0.59) | 3.40 (1.61) | 0.97 (0.31) | 2.18 (1.20) | 1.10 (0.30) | 1.23 (0.93) |
| HCO_3_ (mmol/L) | 24.5 (1.1) | 25.3 (4.7) | 24.9 (2.2) | 23.7 (4.4) | 24.2 (3.3) | 26.5 (4.3) | 24.2 (1.0) | 26.7 (3.7) |
| Base excess (mmol/L) | 1.0 (1.3) | 1.2 (4.2) | 1.0 (2.1) | -0.9 (4.1) | 0.1 (3.3) | 2.5 (3.5) | -0.3 (1.5) | 2.1 (3.2) |
| *Fluids* |  |  |  |  |  |  |  |  |
| Fluid balance total (mL) | 332 (499) | 875 (447) | 376 (1546) | 1025  (274) | -9 (3628) | 671 (808) | 398 (3664) | 780 (383) |
| Urine output/hr (mL/h) | 313 (144) | 180 (192) | 105 (42) | 103 (67) | 87 (38) | 267 (283) | 130 (26) | 45 (21) |
| *Biochemistry* |  |  |  |  |  |  |  |  |
| Aspartate transaminase (U/L) | 76 (10) | 83 (10) | 74 (8) | 190 (80) | 102 (18) | 305 (190) | 106 (19) | 394 (378) |
| Alanine transaminase (U/L) | 79 (33) | 137 (60) | 78 (35) | 180 (87) | 59 (31) | 107 (60) | 64 (22) | 131 (93) |
| Bilirubin (μmol/L) | 4 (1) | 4 (1) | 5 (1) | 6 (2) | 5 (2) | 5 (2) | 8 (5) | 4 (2) |
| Creatine Kinase (U/L) | 325 (109) | 410 (60) | 380 (101) | 597 (135) | 1365 (1230) | 4437 (6091) | 411 (226) | 2379 (2192) |
| Gamma-glutamyl transferase (U/L) | 38 (11) | 41 (4) | 35 (10) | 51 (10) | 33 (7) | 63 (31) | 30 (7) | 51 (18) |
| Creatinine (μmol/L) | 0.06 (0.01) | 0.07 (0.01) | 0.06 (0.01) | 0.07 (0.01) | 0.07 (0.008) | 0.07 (0.005) | 0.07 (0.008) | 0.07 (0) |
| *Hematology* |  |  |  |  |  |  |  |  |
| Hemoglobin (g/L) | 97 (9) | 70 (47) | 107 (30) | 92 (61) | 84 (12) | 89 (13) | 84 (13) | 85 (12) |
| White blood cells (10^9^/L) | 4.5 (2.6) | 2.2 (1.0) | 4.3 (2.9) | 2.1 (0.9) | 7.2 (2.9) | 5.2 (3.5) | 5.1 (3.5) | 2.1 (0.6) |
| Neutrophils (% of total white blood cells) | 59.0 (15.0) | 44.3 (25.9) | 58.0 (8.2) | 43.5 (30.1) | 84.5 (7.0) | 77.3 (5.0) | 74.0 (12.4) | 50.3 (16.6) |
| Lymphocytes (% of total white blood cells) | 31.5 (8.6) | 53.3 (27.4) | 40.5 (9.3) | 29.4 (26.6) | 12.5 (6.2) | 16.3 (1.2) | 18.5 (6.6) | 41.0 (14.1) |
| Platelets (10^9^/L) | 288 (25) | 274 (53) | 288 (25) | 264 (45) | 245 (110) | 300 (56) | 300 (0) | 259 (83) |
| *Catecholamines* |  |  |  |  |  |  |  |  |
| Metanephrine (pmol/L) | 298 (174) | 377 (506) | 3379 (6414) | 12045 (5806) | 146 (49) | 568 (539) | 124 (75) | 370 (485) |
| Normetanephrine (pmol/L) | 1538 (1115) | 2265 (2874) | 2056 (1315) | 16950 (13034) | 1599 (2020) | 5783 (4789) | 2677 (3939) | 5156 (3427) |

**Table S3.** Hemodynamic and ventilatory parameters, blood results and vasoactive use during the course of the study at stabilisation (one hour following completion of all instrumentation procedures), T0 (successful weaning from CPB), T1, T3 and T6 in recipient animals. All values are mean (SD). NA – not available; FiO_2_ – inspiratory fraction of oxygen; PEEP – positive end-expiratory pressure; EtCO_2_ – end-tidal carbon dioxide; PaO^2^ – arterial partial pressure of oxygen; PaCO^2^ – arterial partial pressure of carbon dioxide; HCO_3_ – concentration of bicarbonate in arterial blood.

|  | *Stabilisation* | | *1 hr* | | *3 hr* | | *6 hr* | |
| --- | --- | --- | --- | --- | --- | --- | --- | --- |
|  | **SHAM** | **BSD** | **SHAM** | **BSD** | **SHAM** | **BSD** | **SHAM** | **BSD** |
| *Hemodynamics* |  |  |  |  |  |  |  |  |
| Heart rate (bpm) | 100 (17) | 91 (25) | 115 (8) | 105 (22) | 99 (15) | 110 (23) | 102 (1) | 116 (24) |
| Mean arterial pressure (mmHg) | 111 (22) | 109 (7) | 71 (26) | 67 (10) | 76 (13) | 62 (7) | 61 (14) | 62 (14) |
| Central venous pressure (mmHg) | 15 (5) | 12 (3) | 17 (6) | 16 (5) | 16 (3) | 16 (3) | 16 (3) | 16 (3) |
| Vasopressor dependency index (mmHg^-1^) | 0.029 (0.06) | 0 (0) | 0.75 (0.80) | 0.24 (0.18) | 0.56 (0.59) | 0.55 (0.16) | 0.76 (0.83) | 0.71 (0.18) |
| Body temperature (°C) | 37.5 (0.5) | 37.2 (0.8) | 36.8 (1.0) | 36.1 (1.2) | 36.5 (0.6) | 36.6 (1.0) | 36.4 (0.8) | 36.2 (1.7) |
| *Vasoactive use (μg/kg/min)* |  |  |  |  |  |  |  |  |
| Noradrenaline | 0 (0) | 0 (0) | 0.15 (0.15) | 0.12 (0.08) | 0.23 (0.11) | 0.25 (0.18) | 0.24 (0.12) | 0.31 (0.17) |
| Dopamine | 2.3 (4.6) | 0 (0) | 2.3 (4.6) | 0.19 (0.37) | 0 (0) | 0.19 (0.37) | 0 (0) | 0.25 (0.49) |
| Adrenaline | 0 (0) | 0 (0) | 0.22 (0.24) | 0.04 (0.05) | 0.14 (0.20) | 0.09 (0.11) | 0.14 (0.20) | 0.13 (0.15) |
| *Ventilation* |  |  |  |  |  |  |  |  |
| FiO_2_ (%) | 35 (24) | 45 (6) | 45 (6) | 48 (10) | 40 (0) | 43 (5) | 40 (0) | 43 (5) |
| Minute volume (L/min) | 8.5 (2.3) | 7.8 (1.6) | 10.9 (10.1) | 6.4 (1.4) | 14.5 (15.2) | 6.8 (2.8) | 14.8 (15.8) | 7.3 (2.7) |
| PEEP (cm H_2_O) | 8.3 (2.4) | 7.3 (2.9) | 9.0 (1.2) | 8.0 (1.6) | 8.7 (1.2) | 8.8 (2.2) | 9.3 (1.2) | 8.5 (1.9) |
| EtCO_2_ (mmHg) | 39.3 (6.5) | 41.3 (10.5) | 33.0 (4.1) | 35.0 (5.2) | 36.0 (5.3) | 33.3 (5.7) | 35.3 (2.5) | 31.3 (4.1) |
| *Arterial Blood gases* |  |  |  |  |  |  |  |  |
| pH | 7.46 (0.03) | 7.43 (0.09) | 7.35 (0.11) | 7.19 (0.06) | 7.33 (0.14) | 7.17 (0.08) | 7.27 (0.15) | 7.22 (0.17) |
| PaO_2_ (mmHg) | 252 (36) | 187 (67) | 172 (78) | 116 (33) | 116 (39) | 100 (32) | 108 (38) | 115 (23) |
| PaCO_2_ (mmHg) | 38 (3) | 37 (7) | 37 (5) | 42 (7) | 37 (8) | 42 (4) | 41 (8) | 35 (6) |
| Lactate (mmol/L) | 0.9 (0.3) | 0.8 (0.3) | 5.3 (1.8) | 6.2 (1.1) | 6.6 (4.2) | 9.1 (2.4) | 7.6 (5.9) | 11.3 (5.8) |
| HCO_3_ (mmol/L) | 26.5 (2.0) | 23.6 (2.4) | 19.2 (4.6) | 15.8 (3.3) | 17.8 (4.7) | 13.9 (1.9) | 12.9 (NA) | 13.4 (4.2) |
| Base excess (mmol/L) | 2.8 (2.0) | 0.1 (2.7) | -4.8 (5.0) | -11.0 (3.9) | -5.9 (5.8) | -11.8 (2.9) | -8.4 (8.3) | -12.3 (5.9) |
| *Fluids* |  |  |  |  |  |  |  |  |
| Fluid balance total (mL) | 316 (588) | 482 (563) | 3713 (3045) | 1372 (1038) | 3706 (3184) | 2800 (1696) | 4320 (3036) | 4201 (3170) |
| Urine output/hr (mL) | 223 (182) | 202 (139) | 130 (147) | 40 (57) | 35 (18) | 33 (35) | 45 (30) | 40 (43) |
| *Biochemistry* |  |  |  |  |  |  |  |  |
| Aspartate transaminase (U/L) | 76 (10) | 96 (58) | 740 (483) | 347 (148) | 632 (476) | 370 (119) | 536 (263) | 389 (156) |
| Alanine transaminase (U/L) | 104 (32) | 121 (66) | 125 (53) | 135 (94) | 137 (101) | 150 (129) | 114 (70) | 165 (173) |
| Bilirubin (μmol/L) | 3 (1) | 5 (4) | 4 (4) | 4 (3) | 4 (5) | 3 (4) | 14 (9) | 4 (4) |
| Creatine Kinase (U/L) | 147 (66) | 122 (52) | 4303 (434) | 3539 (1473) | 4385 (430) | 3636 (1464) | 4572 (841) | 4424 (2033) |
| Gamma-glutamyl transferase (U/L) | 38 (5) | 46 (14) | 67 (63) | 43 (10) | 77 (85) | 44 (11) | 59 (51) | 40 (5) |
| Creatinine (μmol/L) | 0.06 (0.007) | 0.07 (0.006) | 0.06 (0.006) | 0.08 (0.002) | 0.06 (0.005) | 0.08 (0.004) | 0.06 (0.004) | 0.08 (0.004) |
| *Hematology* |  |  |  |  |  |  |  |  |
| Hemoglobin (g/L) | 96 (6) | 97 (8) | 98 (6) | 67 (45) | 103 (4) | 74 (50) | 106 (11) | 78 (53) |
| White blood cells (10^9^/L) | 5.2 (2.4) | 3.8 (2.6) | 2.5 (1.2) | 2.8 (1.1) | 2.7 (1.0) | 3.9 (2.3) | 3.2 (1.1) | 5.6 (3.8) |
| Neutrophils (% of total white blood cells) | 63.0 (18.5) | 39.0 (28.4) | 47.3 (34.7) | 38.3 (6.7) | 52.0 (35.9) | 43.3 (4.6) | 55.0 (28.0) | 53.8 (3.3) |
| Lymphocytes (% of total white blood cells) | 35.0 (19.7) | 56.3 (27.8) | 44.3 (32.5) | 46.0 (8.1) | 38.7 (32.6) | 39.0 (4.3) | 36.7 (29.6) | 30.8 (7.9) |
| Platelets (10^9^/L) | 287 (18) | 324 (29) | 191 (103) | 196 (62) | 261 (55) | 184 (135) | 260 (57) | 122 (67) |
| *Catecholamines* |  |  |  |  |  |  |  |  |
| Metanephrine (pmol/L) | 162 (49) | 122 (67) | 10109 (15484) | 5295 (3654) | 13375 (20499) | 7003 (6274) | 11608 (16877) | 8993 (7325) |
| Normetanephrine (pmol/L) | 691 (321) | 614 (132) | 14753 (13091) | 22195 (19105) | 19423 (17917) | 15058 (10177) | 27333 (20648) | 21085 (13282) |

**Table S4.** P values from multiple comparisons test for parameter estimates among Sham and BSD donors at each time point for donor heart rate (HR), plasma metanephrines (Met.), mean arterial pressure (MAP), vasopressor dependency index (VDI), blood lactate, minute volume and fluid balance. B – baseline, ST – stabilisation, T0 – confirmation of BSD, T1-T24 – 1-24 hours post-BSD confirmation.

|  | HR | Plasma Met. | MAP | VDI | Lactate | Minute volume | Fluid balance |
| --- | --- | --- | --- | --- | --- | --- | --- |
| B | 0.75 | 0.43 | 0.05 | 0.9 | 0.36 | 0.48 | 0.27 |
| ST | 0.81 | 0.14 | 0.24 | 0.36 | 0.79 | 0.013 | 0.49 |
| T0 | 0.22 | 0.092 | <0.001 | 0.03 | 0.16 | 0.031 | 0.51 |
| T1 | 0.77 | 0.58 | 0.006 | 0.012 | 0.069 | 0.008 | 0.56 |
| T3 | 0.76 | 0.77 | 0.14 | 0.034 | 0.12 | <0.001 | 0.84 |
| T6 | 0.91 | 0.74 | 0.31 | 0.16 | 0.12 | <0.001 | 0.78 |
| T9 | 0.43 | 0.3 | 0.13 | 0.3 | 0.45 | <0.001 | 0.76 |
| T12 | 0.74 | 0.15 | 0.41 | 0.42 | 0.29 | 0.001 | 0.77 |
| T15 | 0.94 | - | 0.19 | 0.45 | 0.63 | 0.088 | 0.98 |
| T18 | 0.85 | 0.17 | 0.51 | 0.47 | 0.64 | 0.15 | 0.92 |
| T21 | 0.97 | - | 0.13 | 0.41 | 0.63 | 0.31 | 0.84 |
| T24 | 0.58 | 0.34 | 0.076 | 0.82 | 0.87 | 0. | 0.87 |
